# Responsiveness of the Oldenburg Burnout Inventory for Medical Students and Predictors of Sustained Burnout during Clinical Clerkships: A Five‐Wave Longitudinal Cohort Study

**DOI:** 10.1111/jep.70500

**Published:** 2026-06-16

**Authors:** Takafumi Watanabe, Osamu Takakuwa, Tatsuo Akechi

**Affiliations:** ^1^ Department of Psychiatry Nagoya City University Graduate School of Medical Sciences Nagoya Japan; ^2^ Department of Medical Education Nagoya City University Graduate School of Medical Sciences Nagoya Japan

**Keywords:** burnout, clinical clerkships, longitudinal cohort, medical students, Oldenburg burnout inventory, responsiveness

## Abstract

**Rationale:**

Robust outcome measurement is central to evaluating clinical training environments and providing timely support to medical students. Burnout during clinical clerkships is common; however, evidence of the capability of instruments to monitor sustained risks and detect meaningful changes remains limited.

**Aims and Objectives:**

To evaluate the responsiveness of the 11‐item Oldenburg Burnout Inventory–Medical Student version (OLBI‐MS‐11) and examine the baseline predictors of individuals' sustained burnout during their clerkships.

**Methods:**

We examined within‐person change correlations between the OLBI‐MS‐11 and domain‐matched anchors (Maslach Burnout Inventory–General Survey [MBI‐GS] subscales) in a five‐wave longitudinal cohort study of Japanese medical students (*N* = 162). We assessed the instrument's discrimination of MBI‐defined burnout caseness and analysed associations between burnout trajectories and clinically relevant constructs, including psychological flexibility, depressive symptoms, perceived stigma, mistreatment, and absence of clerkship.

**Results:**

The OLBI‐MS‐11 demonstrated moderate, domain‐concordant correlations in within‐person changes with the MBI‐GS subscales across adjacent time points and maintained a stable discrimination of burnout caseness (AUC ≈ 0.82–0.84). Changes in burnout were associated with concurrent changes in psychological flexibility and depressive symptoms. Higher baseline psychological flexibility was associated with a reduced risk of sustained burnout, whereas mistreatment predicted persistent burnout.

**Conclusions:**

The OLBI‐MS‐11 showed responsiveness and discriminative validity in clinical clerkships. Its use may facilitate the longitudinal monitoring of medical students' well‐being and inform the identification of sustained burnout risk. Continuous‐change metrics may provide a more nuanced evaluation of individual trajectories than dichotomous classifications alone.

**Abbreviations**
AUC: area under the ROC curve
CI: confidence interval
CY: Cynicism (MBI‐GS subscale)
Dis: disengagement (OLBI‐MS subscale)
EMMs: estimated marginal means
ES: effect size
EX: exhaustion (MBI‐GS subscale)
Exh: exhaustion (OLBI‐MS subscale)
FDR: false discovery rate
HC3: heteroskedasticity‐consistent (HC3) standard errors
ICD‐11: International Classification of Diseases, 11th Revision
IQR: interquartile range
LMM: linear mixed model
MBI‐GS: Maslach Burnout Inventory—general survey
MCID: minimal clinically important difference
MID: minimal important difference
MNAR: missing not at random
NPV: negative predictive value
OLS: ordinary least squares
OLBI‐MS‐11: 11‐item Oldenburg Burnout Inventory—Medical Student
OLBI‐MS‐16: 16‐item Oldenburg Burnout Inventory—Medical Student
OSCE: Objective Structured Clinical Examination
PDDS: Perceived Devaluation–Discrimination Scale
PE: Professional Efficacy (MBI‐GS subscale)
PHQ‐9: Patient Health Questionnaire‐9
PPV: positive predictive value
RCI: Reliable Change Index
ROC: receiver operating characteristic
SD: standard deviation
SE: standard error
SRM: standardized response mean
VIF: variance inflation factor
VQ‐O: Valuing Questionnaire—Obstruction
VQ‐P: Valuing Questionnaire—Progress
WAAQ: Work‐related Acceptance and Action Questionnaire

## 1 Introduction

Burnout is defined in the World Health Organization's International Classification of Diseases, 11th Revision (ICD‐11), as an occupational phenomenon characterised by exhaustion, increased mental distance or cynicism, and reduced professional efficacy [1]. Burnout is associated with poorer health and functioning, diminished quality of care, absenteeism, and attrition among clinicians; thus, it is a key workforce‐and‐patient‐safety concern [2, 3]. Burnout is also common among medical students and often increases during their clinical years [4, 5, 6]; thus, their clerkships are identified as a critical period for stress‐related health outcomes. In Japan, cross‐sectional data from students in clinical clerkships suggest comparable levels of burnout [7]. Factors associated with higher burnout include depressive and anxiety symptoms, lower psychological flexibility—the capacity to pursue valued goals without avoiding uncomfortable internal experiences [7, 8, 9] — perceived stigma [10, 11], and mistreatment during training [12, 13]. Ongoing reforms towards more team‐based clerkships in Japan further increase the need for brief, valid tools with which burnout can be monitored and educational and support interventions can be informed [14].

Burnout and depression are closely intertwined [15], despite being distinct constructs [16]. Burnout is typically conceptualised as a work‐ or study‐related syndrome characterised by exhaustion and disengagement, whereas depression is a broader clinical condition encompassing affective, cognitive, and somatic symptoms pervading different contexts. An empirical overlap between these constructs has been reported, which may have influenced the observed associations. The prevalence of diagnosable mental disorders among physicians appears similar to that in the general population; suicide mortality is substantially higher among physicians, indicating the possibility of the delayed detection and treatment [17, 18]. Stigma of mental illness and help‐seeking may contribute to such delays when severe distress is framed solely as ‘burnout’ [10, 11, 19], while psychological flexibility has emerged as a modifiable, process‐level resource targeted by Acceptance and Commitment Training that can reduce burnout and stigma in medical trainees [20, 21, 22, 23].

The Maslach Burnout Inventory–General Survey (MBI‐GS) is widely used in medical‐ education research but has limitations in longitudinal monitoring. In particular, the professional efficacy subscale is positively worded in the scale, whereas exhaustion and cynicism are negatively worded in it, which can introduce method variance and inconsistent associations [24, 25]. The Oldenburg Burnout Inventory (OLBI) was developed to address some of these issues by assessing exhaustion and disengagement using balanced and positively and negatively worded items and a broader cognitive and physical framing of exhaustion [26, 27]. The medical student version (OLBI‐MS) has been used in large U.S. surveys and student samples [28, 29, 30]. Building on our previous measurement work, we focus here on the 11‐item short form (OLBI‐MS‐11), whose structural validity and reliability in Japanese clinical clerkships are reported elsewhere [31]. In brief, OLBI‐MS‐11 preserves the intended two‐factor structure (exhaustion and disengagement) and shows acceptable internal consistency and measurement invariance, and the full psychometric details are reported in a separate preprint.

Despite the extensive use of burnout measures in medical education, the responsiveness of the OLBI‐MS—its ability to detect within‐person changes over time—has rarely been examined, and, to our knowledge, has not been studied in Japanese clinical clerkships. Most studies on medical students are cross‐sectional and describe prevalence and correlates rather than trajectories of change [5, 6, 29, 30, 32, 33]. Existing longitudinal research tends to focus more on broader well‐being or contextual factors than on the responsiveness of specific instruments and has largely been conducted outside Japan. Moreover, although lower psychological flexibility, perceived stigma, and mistreatment have been consistently related to burnout in cross‐sectional analyses, the baseline predictors of sustained burnout during clerkship remain poorly understood in the Japanese context. Work on internal structure also points to the limitations of the original 16‐item OLBI‐MS and supports the use of a shorter, structurally coherent form, but has not addressed responsiveness [34]. Administrative indicators such as clerkship absenteeism provide an external, behaviourally meaningful marker of functioning that is directly relevant to educational continuity. However, no study has examined whether within‐person changes in OLBI‐MS scores reflect adjacent interval changes in absenteeism among medical students.

## 2 Objectives

We conducted a five‐wave longitudinal cohort study of Japanese medical students in clinical clerkships to evaluate the responsiveness of the OLBI‐MS subscales and identify baseline predictors of subsequent burnout levels. First, we examined whether within‐person changes in the OLBI‐MS Exhaustion and Disengagement scores were associated with contemporaneous changes in burnout measures, psychological flexibility, depressive symptoms, perceived stigma, mistreatment, and clerkship absenteeism. Second, we tested whether baseline characteristics (age, sex, psychological flexibility, stigma, and mistreatment frequency) predicted the mean OLBI‐MS scores across the clerkship period. We focused on psychological flexibility, stigma, and mistreatment because they are potentially modifiable intra‐ and interpersonal processes that have already been targeted in emerging interventions for medical students.

## 3 Methods

### 3.1 Design, Setting and Participants

We conducted a five‐wave longitudinal cohort study on medical students during their clinical clerkships at Nagoya City University Hospital in Japan. Two overlapping academic cohorts were followed: the 2023 cohort (whose data were collected in January 2023–September 2024) and the 2024 cohort (whose data were collected in December 2023–September 2025). Wave 1 (baseline) occurred immediately before clerkships, and Waves 2–4 took place at approximately 6‐month intervals during clerkships. Wave 5 occurred immediately after clerkship completion. In each wave, the students completed self‐administered questionnaires.

Clerkships rotated across internal medicine and surgical subspecialties and other core departments (e.g. paediatrics, psychiatry, anaesthesiology, radiology, and pathology). The 2023 cohort completed 1–4‐week rotations and one 4‐week elective. The 2024 cohort had 1–2‐week rotations in all departments, followed by four 4‐week specialty or elective blocks. The cohort was designed to examine the responsiveness of burnout measures and the correlates of burnout across clerkship years.

Eligible participants were fifth‐ and sixth‐year medical students undertaking clerkships. In Wave 1, the number of students who consented to participate was 164. Two students were excluded because they were not in the clerkship stream (one repeat‐year student and one visiting trainee), leaving a final analytical cohort of 162 students. The target sample size (*n* = 164) was set based on the COSMIN guidance for responsiveness (≥ 100 participants) with an allowance for missing data; the analytic sample comprised 162 students. No exclusions were made based on health or academic performance. For responsiveness analyses, adjacent wave pairs were included if both waves had non‐missing scores. For predictor analyses, students required baseline covariates and at least one OLBI‐MS observation during follow‐up.

### 3.2 Ethics Approval and Consent to Participate

This study was approved by the Nagoya City University Institutional Review Board (approval date: 2 Nov 2022) and registered in the UMIN Clinical Trials Registry (UMIN000048742; registration date: 24 Aug 2022). This study was conducted in accordance with the principles of the Declaration of Helsinki. All participants received written and verbal information about the study before starting their clinical clerkship and were informed that non‐participation would not affect their grades and that their privacy would be protected. Students were given time to consider their participation, and those who agreed provided their informed consent via the online survey platform.

### 3.3 Measures

#### 3.3.1 Burnout (Primary Measure)

Burnout was assessed using the Japanese 11‐item OLBI‐MS short form (OLBI‐MS‐11), which includes exhaustion (Exh; five items) and disengagement (Dis; 6 items). Items were rated on a 4‐point Likert scale (1–4); subscale scores were calculated as means, with higher values indicating greater burnout. OLBI‐MS was administered in all waves (W1–W5). Although a 16‐item OLBI‐MS exists, we prespecified the validated 11‐item form as the primary instrument to minimise the respondent burden. The structural validity and reliability of the Japanese 11‐item form have been reported elsewhere [31]. Pre‐specified sensitivity analyses using the 16‐item form are summarised below and detailed in the Supporting Information.

#### 3.3.2 Burnout Anchor

The MBI‐GS served as an external criterion for responsiveness. It comprises exhaustion (EX), cynicism (CY), and Professional Efficacy (PE) subscales [25, 35]. Items are rated on a 7‐point Likert scale (0–6), and subscales are scored as means, with higher EX and CY indicating greater burnout and higher PE indicating better functioning. The MBI‐GS was administered to all waves. For change–change responsiveness, the primary anchors were MBI‐EX for the OLBI‐Exh and MBI‐CY for the OLBI‐Dis, reflecting domain‐concordant pairing. MBI‐PE was retained as a secondary anchor (expected negative association) for complementary analyses. Predefined cutoffs for caseness (MBI‐EX > 4.0, MBI‐CY > 2.6) are described in the statistical analysis section.

#### 3.3.3 Psychological Flexibility (Acceptance and Valued Action) and Other Correlates

Work‐related psychological flexibility was measured using the 7‐item Work‐related Acceptance and Action Questionnaire (WAAQ; higher scores indicate greater flexibility) [36, 37]. Valued action was assessed using the Valuing Questionnaire (VQ), which includes the Progress (VQ‐P) and Obstruction (VQ‐O) subscales [38, 39]. Perceived stigma towards mental illness was assessed with the 12‐item Perceived Devaluation–Discrimination Scale (PDDS; higher scores indicate stronger perceived stigma) [40, 41]. Depressive symptoms were assessed with the Patient Health Questionnaire‐9 (PHQ‐9; higher scores indicate more severe symptoms) [42, 43]. Mistreatment was assessed with a single item meant for eliciting information on the frequency of mistreatment by faculty or residents (‘never’, ‘once or twice’, ‘several times’, ‘many times’) [12]. These measures were administered at W1–W5.

#### 3.3.4 Clerkship Absenteeism (Behavioural Indicator) and Baseline Covariates

Administrative counts for absent days were obtained from the education office for each adjacent interval (W2–W1, W3–W2, W4–W3, and W5–W4). Thus, absenteeism served as an interval‐level behavioural outcome spanning W1 to W5. At baseline, we recorded age, sex, and contextual variables (living situation, alcohol use, extracurricular and part‐time activities, and prior enrolment in another faculty).

### 3.4 Procedures and Outcomes

In each wave, students completed the OLBI‐MS, MBI‐GS, WAAQ, VQ, PHQ‐9, PDDS, and mistreatment items. For each adjacent interval, clerkship absenteeism (days) was extracted from academic records with the participants' consent. To help mitigate the common method bias inherent in self‐report measures, we triangulated the questionnaire data with an administrative behavioural indicator (clerkship absence days) and used robust inference procedures where appropriate.

The primary endpoint was criterion‐based responsiveness of the OLBI‐MS, defined as within‐person adjacent change–change associations between ΔOLBI‐Exh and ΔMBI‐EX, and between ΔOLBI‐Dis and ΔMBI‐CY. We also evaluated level‐based discrimination by estimating the ability of contemporaneous OLBI‐MS subscale scores to classify MBI‐defined caseness (MBI‐EX > 4.0, MBI‐CY > 2.6) at each wave and in pooled analyses using the area under the receiver operating characteristic (ROC) curve (AUC).

Secondary endpoints assessed convergent responsiveness to changes in professional efficacy (MBI‐PE), psychological flexibility (WAAQ, VQ‐P, VQ‐O), depressive symptoms (PHQ‐9), perceived stigma (PDDS), mistreatment frequency, and clerkship absenteeism. An exploratory analysis was performed to examine whether baseline age, sex, WAAQ, PDDS, and mistreatment predicted person‐mean OLBI‐Exh and OLBI‐Dis scores over the study period.

### 3.5 Statistical Analysis

All the instruments were scored according to the manual. Reverse‐keyed items were recoded so that higher values reflected greater construct severity (e.g., higher Exh/Dis, PHQ‐9, PDDS, VQ‐O), whereas higher WAAQ and VQ‐P scores indicated better functioning. Adjacent within‐person change scores were computed as Δ = W(t) − W(t − 1). Continuous baseline predictors were standardised to *z*‐scores, and sex was modelled as a categorical factor. Analysis‐specific sample sizes have also been reported.

Following COSMIN guidance on responsiveness [44], primary responsiveness was quantified using Pearson correlations between ΔOLBI‐Exh and ΔMBI‐EX and between ΔOLBI‐Dis and ΔMBI‐CY, with 95% confidence intervals obtained via Fisher's *z* transformation [45]. Correlations were summarised across adjacent wave pairs using pairwise‐complete observations [46, 47].

For level‐based discrimination, ROC curves were estimated for OLBI‐Exh against MBI‐EX > 4.0 and for OLBI‐Dis against MBI‐CY > 2.6, both by wave and pooled across waves. We report AUC estimates with DeLong 95% confidence intervals [48] and describe the sensitivity and specificity at Youden optimal thresholds [49].

Convergent responsiveness was assessed using Pearson correlations between ΔOLBI subscales and changes in MBI‐PE, WAAQ, VQ‐P, VQ‐O, PHQ‐9, PDDS, and mistreatment and absenteeism. Distribution‐based indices included the standardised response mean (SRM; mean Δ/SD of Δ) and a Cohen‐type effect size (ES; mean Δ/SD at baseline) for each interval [50, 51]. Anchor‐based responsiveness used MBI cut‐offs to classify adjacent‐interval change as ‘improved’, ‘worsened’, or ‘stable’; we summarised Δ within categories and evaluated the ability of ΔOLBI subscales to discriminate improved versus non‐improved using ROC and AUC.

For the exploratory prediction of sustained burnout, we fitted ordinary least squares regression models with heteroskedasticity‐consistent (HC3) standard errors [52, 53]. Person‐mean OLBI‐Exh and OLBI‐Dis scores (averaged across available waves) were regressed on baseline age, sex, WAAQ, PDDS, and mistreatment. A two‐sided *α* = 0.05 guided inference, with emphasis on effect sizes and confidence intervals.

#### 3.5.1 Sensitivity Analyses and Descriptive Trajectories

To assess robustness, we conducted sensitivity analyses stratified by academic cohort (2023 vs. 2024) and OLBI‐MS item sets (11‐ vs. 16‐item versions). For cohort sensitivity, primary Δ–Δ correlations and ROC/AUC estimates were recomputed within cohorts. For instrument‐length sensitivity, responsiveness, and discrimination analyses were repeated for the 16‐item OLBI‐MS.

We also summarised wave‐wise trajectories for key indicators (OLBI‐MS subscales, MBI‐GS subscales, mistreatment, and absence) using linear mixed‐effects models with random intercepts for participants [54]. Type III tests with Satterthwaite degrees of freedom were obtained using the lmerTest, and pairwise post hoc comparisons were based on model‐derived estimated marginal means with Benjamini–Hochberg false discovery rate adjustment [55, 56, 57]. Full outputs are reported in the Supporting Information.

#### 3.5.2 Missing Data

All available observations were used for analyses; change‐change correlations and ROC/AUC analyses were based on pairwise complete data, whereas regression models were used on complete cases for baseline covariates, and no imputation was performed.

## 4 Results

### 4.1 Cohort Characteristics and Data Structure

At baseline, 162 students (2023 cohort *n* = 88; 2024 cohort *n* = 74) formed the analytical sample. The cohorts were similar in terms of demographics and most of the study variables (Table 1). There were no between‐cohort differences in age, sex, living situation, alcohol use, extracurricular or part‐time activities, PHQ‐9, WAAQ, OLBI‐MS‐11 Exh or Dis, or MBI‐EX. The 2024 cohort had higher MBI‐CY and MBI‐PE. Across the five waves, 765 observations contributed to level‐based ROC analyses, and 603 adjacent wave pairs contributed to change–change correlations. Baseline predictor analysis included 162 participants.

**Table 1:** Baseline characteristics by cohort (Wave 1).

Variable	Total number (*n* = 162)	2023 cohort (*n* = 88)	2024 cohort (*n* = 74)	*p*‐value
		**Number (%)**		
Gender (men)	96 (59.3)	52 (59.1)	44 (59.5)	1.000
Gender (women)	66 (40.7)	36 (40.9)	30 (40.5)	
Alcohol consumption more than 3 times/week	22 (13.6)	12 (13.6)	10 (13.5)	1.000
Participation in an extracurricular activity	117 (72.2)	68 (77.3)	49 (66.2)	0.159
Presence of housemates	85 (52.5)	49 (55.7)	36 (48.6)	0.431
History of enrolment in other faculties	23 (14.2)	10 (11.4)	13 (17.6)	0.270
Presence of a part‐time job	129 (79.6)	71 (80.7)	58 (78.4)	0.845
Spouse	2 (1.2)	2 (2.3)	0 (0.0)	0.501
		**Median [IQR]**		
Age	23.00 [22.00, 23.00]	23.00 [22.00, 23.00]	22.00 [22.00, 23.00]	0.413
MBI‐EX	3.00 [1.80, 4.15]	2.90 [1.20, 4.00]	3.10 [1.80, 4.20]	0.179
MBI‐CY	1.40 [0.60, 2.40]	1.10 [0.35, 2.20]	1.60 [1.00, 2.55]	0.012
MBI‐PE	2.33 [1.33, 3.46]	2.33 [1.00, 2.87]	2.92 [1.50, 3.83]	0.006
OLBI‐MS‐11 Exh	2.40 [2.00, 2.80]	2.40 [2.00, 2.80]	2.60 [2.00, 2.80]	0.280
OLBI‐MS‐11 Dis	2.00 [1.83, 2.33]	2.00 [1.83, 2.33]	2.17 [1.83, 2.50]	0.241
PHQ‐9	4.00 [2.00, 7.75]	4.00 [1.00, 8.00]	5.00 [2.00, 7.00]	0.645
VQ‐P	19.00 [15.00, 22.00]	17.00 [15.00, 22.00]	20.00 [16.00, 23.00]	0.069
VQ‐O	16.00 [13.00, 19.00]	16.00 [13.00, 19.00]	17.00 [14.00, 19.00]	0.250
WAAQ	28.00 [22.00, 30.00]	28.00 [23.00, 30.25]	28.00 [22.00, 30.00]	0.913
PDDS	24.00 [20.00, 26.00]	23.00 [19.75, 26.00]	24.00 [21.00, 26.00]	0.189
Mistreatment	1.00 [1.00, 1.00]	1.00 [1.00, 1.25]	1.00 [1.00, 1.00]	0.956

*Note:* Values are presented as *n* (%) for categorical variables and median [IQR] for continuous variables. *p* values are from *χ* ^2^ tests for categorical variables and Wilcoxon rank‐sum tests for continuous variables; all tests were two‐sided.

Abbreviations: Dis, disengagement; Exh, exhaustion; IQR, interquartile range; MBI‐CY, Maslach Burnout Inventory—Cynicism; MBI‐EX, Maslach Burnout Inventory—exhaustion; MBI‐GS, Maslach Burnout Inventory—General Survey; MBI‐PE, Maslach Burnout Inventory—professional efficacy; OLBI‐MS‐11, Oldenburg Burnout Inventory‐Medical Student (11‐item short form); PDDS, Perceived Devaluation–Discrimination Scale; PHQ‐9, Patient Health Questionnaire‐9; VQ‐O, Valuing Questionnaire—Obstruction; VQ‐P, Valuing Questionnaire—Progress; WAAQ, Work‐related Acceptance and Action Questionnaire.

### 4.2 Criterion‐Based Responsiveness

**Figure 1:**
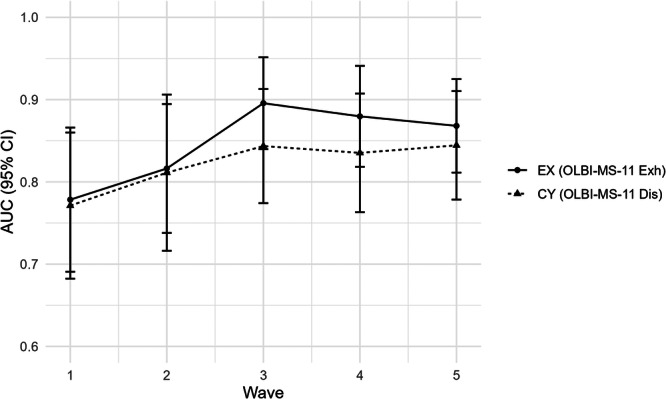
Wave‐specific AUCs for classifying MBI caseness from OLBI‐MS‐11. Points show AUC estimates with 95% confidence intervals for each wave; the solid line denotes exhaustion (MBI‐EX > 4.0) and the dashed line denotes disengagement (MBI‐CY > 2.6).

Within‐person changes in the OLBI‐MS‐11 were aligned with concurrent changes in domain‐matched MBI‐GS subscales. ΔOLBI‐Exh showed moderate correlations with MBI‐EX and ΔOLBI‐Dis with ΔMBI‐CY (both *p* < 0.001; Table 2). At the level of concurrent discrimination, OLBI‐MS‐11 subscale scores classified MBI‐defined caseness with good accuracy: pooled AUCs were approximately 0.82–0.84, and wave‐wise AUCs remained in the ‘good’ range (Table 3; Figure 1). Youden‐optimal thresholds in pooled data were 2.50 for OLBI‐Exh (sensitivity, 87%; specificity, 67%; positive predictive value, 45%; negative predictive value, 94%) and 2.42 for OLBI‐Dis (sensitivity, 71%; specificity, 76%; positive predictive value, 46%; negative predictive value, 90%). These operating points are sample‐specific and are provided for an interpretive context rather than the recommended screening cutoffs.

**Table 2:** Adjacent‐interval change–change correlations between OLBI‐MS subscales and domain‐concordant MBI‐GS anchors (primary responsiveness analysis).

Pair *	*n*	Pearson r ** [95% CI]	Spearman ρ *** [95% CI]	*p*‐value
ΔOLBI‐MS‐11 Exh ↔ ΔMBI‐EX	603	0.347 [0.274, 0.415]	0.371 [0.300, 0.438]	< 0.001
ΔOLBI‐MS‐11 Dis ↔ ΔMBI‐CY	603	0.349 [0.277, 0.417]	0.343 [0.271, 0.412]	< 0.001

*Note:* Δ denotes within‐person adjacent change computed as Δ = W(t) − W(t − 1) for intervals W2–W1, W3–W2, W4–W3, and W5–W4. Primary estimates are Pearson's r with 95% CIs derived via Fisher's z transformation; Spearman's ρ is provided as a sensitivity analysis. Two‐sided tests, α = 0.05; no multiplicity adjustment. *n* reflects the complete pairwise observations pooled across all adjacent intervals. Domain‐concordant anchors: ΔOLBI‐MS‐11 Exh with ΔMBI‐EX; ΔOLBI‐MS‐11 Dis with ΔMBI‐CY. Abbreviations: CI, confidence interval; Dis, disengagement; Exh, exhaustion; MBI‐CY, Maslach Burnout Inventory—Cynicism; MBI‐EX, Maslach Burnout Inventory—exhaustion; MBI‐GS, Maslach Burnout Inventory—General Survey; OLBI‐MS‐11, Oldenburg Burnout Inventory‐Medical Student (11‐item short form).

**Table 3:** Wave‐specific and pooled AUCs for OLBI‐MS‐11 scores classifying concurrent MBI‐GS caseness.

(A) Exh → MBI‐EX				
Wave	*N*	Positives	Negatives	AUC [95% CI]
1	162	32	130	0.771 [0.682, 0.860]
2	149	27	122	0.811 [0.716, 0.906]
3	151	39	112	0.844 [0.774, 0.913]
4	153	34	119	0.835 [0.763, 0.907]
5	150	37	113	0.844 [0.778, 0.910]
Pooled	765	169	596	0.821 [0.787, 0.855]

(B) Dis → MBI‐CY				
Wave	*N*	Positives	Negatives	AUC [95% CI]
1	162	41	121	0.778 [0.691, 0.866]
2	149	41	108	0.816 [0.738, 0.895]
3	151	36	115	0.896 [0.840, 0.951]
4	153	33	120	0.880 [0.818, 0.941]
5	150	27	123	0.868 [0.811, 0.925]
Pooled	765	178	587	0.846 [0.815, 0.878]

(A) Exh → MBI‐EX, (B) Dis → MBI‐CY. Caseness thresholds were pre‐specified from prior literature and are detailed in the statistical analysis: MBI‐EX > 4.0, and MBI‐CY > 2.6. For each wave and pooled data, contemporaneous ROC curves were estimated for OLBI‐MS‐11 Exh against MBI‐EX, and OLBI‐MS‐11 Dis against MBI‐CY. AUCs were reported with 95% CIs by DeLong, where provided, Youden‐optimal OLBI‐MS‐11 cut‐off points appeared with sensitivity and specificity (descriptive only). “Pooled” aggregates all wave‐level observations. N denotes observations with non‐missing OLBI‐MS‐11 and MBI‐GS in that wave and positives/negatives are counted above or below the panel‐specific MBI threshold. We interpreted AUC ≥ 0.70 as acceptable discrimination. The pooled Youden optimal thresholds (descriptive) were as follows: Exh 2.50, Dis 2.42.

Abbreviations: AUC, area under the curve; CI, confidence interval; Dis, disengagement; Exh, exhaustion; MBI‐CY, Maslach Burnout Inventory—cynicism; MBI‐EX, Maslach Burnout Inventory—exhaustion; MBI‐GS, Maslach Burnout Inventory—General Survey; OLBI‐MS‐11, Oldenburg Burnout Inventory—Medical Students (11‐item short form).

### 4.3 Construct‐Based Responsiveness

As hypothesised, changes in OLBI‐MS‐11 scores showed covariance with external measures (Supplementary Figure S1 and Supplementary Table S1). Increases in depressive symptoms were associated with increases in burnout (PHQ‐9: Exh *r* = 0.270, 95% CI 0.195–0.343; Dis *r* = 0.281, 0.206–0.353; both having *p* < 0.001). Increases in work‐related psychological flexibility were associated with decreases in burnout (WAAQ: Exh *r* = −0.170, −0.247 to −0.092; Dis *r* = −0.177, −0.253 to −0.099; *p* < 0.001). Valued actions showed similar patterns, and changes in stigma and mistreatment showed small domain‐specific associations. PDDS was positively associated with OLBI‐Exh, but not with Dis, whereas mistreatment frequency was positively associated with OLBI‐Dis, but not with Exh. Change‐based absence was not significantly associated with ΔOLBI‐Exh or ΔOLBI‐Dis. In contrast, cumulative absence and the binary indicator of ‘any absence in the interval’ showed small but statistically significant concurrent associations with Dis only (Supplementary Tables S2, S3). As anticipated, changes in MBI‐PE did not show a clear Δ–Δ association with OLBI‐MS‐11.

### 4.4 Distribution‐Based Indices and Descriptive Trajectories

Standardised response means were negative and of moderate magnitude among MBI‐defined ‘Improved’ groups and close to zero among ‘stable/non‐improved’ groups, indicating distribution‐based responsiveness that aligned with anchor‐defined change categories. Using ΔOLBI‐MS‐11 for the classification of MBI‐defined improvement yielded AUCs at 0.66–0.68, with high negative predictive values reflecting the relatively low proportion of improved cases (Supplementary Tables S4, S5).

Interval‐wise standardised response means (SRM) for OLBI‐MS‐11 exhaustion and disengagement are shown in Supplementary Figure S2, illustrating small to moderate changes over successive clerkship intervals that were consistent with the primary responsiveness findings. Across the waves, disengagement on OLBI increased modestly (peaking at W3), whereas exhaustion decreased gradually, in line with the small‐to‐moderate SRMs. Mistreatment increased steadily, and absences peaked mid‐year and declined by W5. Trajectories for the OLBI‐MS and MBI subscales, mistreatment, and absence are summarised in Supplementary Figure S3 and detailed in Supplementary Tables S6, S7 and Supplementary Figure S4. These patterns were broadly similar across the cohorts.

### 4.5 Baseline Predictors of Average OLBI‐MS Across Clerkships

**Figure 2:**
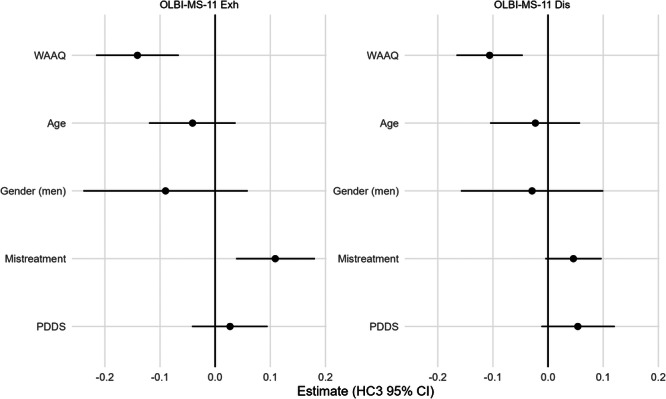
Baseline predictors of person‐mean OLBI‐MS‐11 (HC3‐robust OLS). Points represent cohort‐adjusted coefficients and bars show 95% confidence intervals. Continuous covariates are standardised (*z*‐scores).

The HC3‐robust regression models indicated that higher baseline psychological flexibility predicted lower person‐mean Exh and Dis scores (both *p* < 0.001). Higher baseline mistreatment predicted a higher person‐mean Exh (*p* < 0.01) and was not associated with Dis. Baseline PDDS score, age, and sex were not significant predictors. The modest model fit (Exh *R* ^2^ = 0.179; Dis *R* ^2^ = 0.130) indicates that additional factors were likely to contribute to sustained burnout (Figure 2; Supplementary Table S8 provides the full regression output).

### 4.6 Sensitivity Analyses

Primary Δ–Δ correlations were similar in the 2023 and 2024 cohorts, with no significant between‐cohort differences (Table S9), and AUCs for level‐based discrimination were also comparable across cohorts (Table S10). Distribution‐based indices for the W1–W5 contrast showed small cohort‐specific differences in absolute change; however, these did not affect the criterion‐based conclusions (Table S11). Analyses using the original 16‐item OLBI‐MS also closely paralleled the short‐form findings: ΔOLBI‐16 Exh and ΔOLBI‐16 Dis correlated moderately with ΔMBI‐EX and ΔMBI‐CY; pooled AUCs remained in the good range; distribution‐ and anchor‐based indices showed similar patterns. The complete results for the 16‐item form are provided in Supplementary Tables S12–S14 and Supplementary Figures S3, S4.

## 5 Discussion

This five‐wave longitudinal study examined the responsiveness of the 11‐item OLBI‐MS among Japanese medical students undertaking clinical clerkships. Within‐person changes in the OLBI‐MS‐11 reflected changes in the corresponding MBI‐GS domains, and concurrent OLBI‐MS‐11 scores showed good discrimination of MBI‐defined caseness. OLBI‐MS‐11 changes also covaried in expected directions with psychological flexibility, depressive symptoms, valued action, perceived stigma, and mistreatment and showed interpretable associations with absence during clerkship as a behavioural indicator. Baseline psychological flexibility predicted lower person‐mean exhaustion and disengagement, while higher baseline mistreatment predicted higher exhaustion. These patterns were consistent across academic‐year cohorts and during the use of the 16‐item form, suggesting that OLBI‐MS‐11 demonstrated suitable measurement properties for monitoring and evaluation within clerkships, including the evidence of criterion‐based responsiveness and stable discriminative performance in repeated‐measures contexts. These results situate medical students' burnout during clerkship within the broader psychological and contextual processes that co‐occur with depressive symptoms, perceived stigma, and other contextual factors.

Debate continues on the positively keyed Professional Efficacy subscale of the MBI‐GS, including concerns about method effects and its variable alignment with exhaustion and cynicism [24, 25, 58]. The OLBI framework, in which positively and negatively keyed items are balanced and the cognitive and somatic aspects of exhaustion are captured, offers a pragmatic alternative for repeated monitoring in educational settings in which brief and psychometrically balanced instruments are advantageous [26, 27]. However, most prior studies have emphasised the internal structure and prevalence estimates, and evidence of longitudinal performance within real‐world clerkships has been scarce. Our study extends this evidence base by evaluating change‐tracking, concurrent discrimination, and robustness across cohorts and instrument versions (11‐ vs 16‐item forms). Although the OLBI‐MS‐11 and MBI‐GS are used to assess related constructs, the use of domain‐matched anchors offers a pragmatic reference point for evaluating responsiveness in applied settings.

For educators and programme leaders, concurrent AUCs at 0.82 indicate that OLBI‐MS‐11 may contribute to screening and cohort‐level monitoring [59, 60]. In contrast, Δ‐based AUCs at 0.65 for anchor‐defined improvement suggest that using simple change cut‐offs is insufficient to judge individual progress and that the instrument has relatively limited sensitivity for detecting individual‐level changes. Class imbalance, dependence on MBI cutoffs, and short‐term contextual fluctuations likely contribute to these factors. Where individual‐level decisions are required, it may be preferable to combine raw change scores with a reliable change index and estimates of a minimal important difference derived from both anchor‐based ROC analyses and distribution‐based rules of thumb, thereby integrating statistical reliability with interpretability thresholds [61, 62]. Continuous change metrics and indices such as SRM and ES are useful for evaluating the impact of curricular or support initiatives [50, 51] and, in this cohort, aligned well with anchor‐defined improvement.

The observed wave‐wise trajectories have implications for clerkship design and support. The gradual increase in disengagement, together with a decline in exhaustion and a stable MBI‐CY, suggests that students may shift from acute strain to quieter disengagement as the year progresses. Against a backdrop of rising self‐reported mistreatment and mid‐year peaks in absence and in line with research linking mistreatment during training to burnout and attrition [13], this pattern underlines the value of tracking disengagement explicitly, rather than relying solely on exhaustion‐based measures. Although prior occupational studies have linked exhaustion to sickness absence [63, 64], we found no robust Δ–Δ association between interval absence and exhaustion or disengagement on OLBI and only small level‐based associations for disengagement. In clerkships, absence often reflects timetabling and short‐term constraints as much as distress, and motivational withdrawal captured by disengagement may manifest as reduced engagement, preparation, or participation, which traditional absence metrics do not detect [65].

The prediction analyses highlighted two actionable levers: psychological flexibility and mistreatment. Lower baseline psychological flexibility predicts higher average exhaustion and disengagement, whereas higher baseline mistreatment predicts higher exhaustion. These findings converge with emerging evidence that acceptance and commitment training can enhance psychological flexibility and reduce burnout symptoms among medical trainees [20, 23] and that learner‐directed upstander training may strengthen trainees' capacity to respond to mistreatment and microaggressions [66]. Therefore, within medical education, OLBI‐MS‐11 can serve both as an outcome measure for such interventions and as a monitoring tool to identify cohorts or subgroups at risk. However, modest R^2^ values indicate that broader structural and cultural factors, such as rotation organisation, workload, sleep, team climate, and supervisory support, are likely to play important roles [67, 68]. Therefore, the present models capture only part of the variability in burnout outcomes. From a theoretical perspective, these findings can be situated within the frameworks for analysing job‐demand resources and conservation of resources. In these frameworks, burnout is conceptualised as something that occurs when high and chronic demands are not balanced by sufficient resources and when valued resources are threatened or lost [69, 70, 71]. Psychological flexibility may function as a personal resource that buffers the impact of demanding clerkships, whereas mistreatment represents a social‐structural demand that accelerates resource‐loss spirals, highlighting the need for coordinated individual‐ and system‐level interventions in medical education. Given the conceptual and empirical overlap between burnout and depressive symptoms, these findings should be interpreted with caution, as part of the observed relationships may reflect shared variance between constructs rather than entirely distinct processes [16].

This study has several strengths. It integrates multiple approaches to responsiveness within a five‐wave longitudinal design, combines self‐report scales with an administrative indicator of functioning, and includes robustness checks across cohorts and item sets. The analyses used techniques, including HC3 standard errors, DeLong confidence intervals, and linear mixed models, which are increasingly recommended in educational research.

However, the following limitations should be acknowledged. The single‐site design represents a critical limitation of this study and restricts the generalisability of the findings to other educational settings both within and outside Japan, including institutions with different organisational structures (e.g., public vs. private), curricular models, and cultural contexts. Although the internal validity of the analyses is supported by the longitudinal design and robustness checks, external validity remains uncertain. Therefore, the present findings should be interpreted with caution, and replication across multiple institutions and healthcare systems is required before broader generalisation. Reliance on self‐reported measures introduces a potential response‐related bias. However, the inclusion of administrative‐absence records provides an objective data source that partially mitigates this limitation. Missing data were handled with pairwise deletion for correlational analyses and complete‐case analysis for regression; therefore, missing data that were not at random could not be excluded. Δ‐based ROC analyses were influenced by class imbalance, with the expected trade‐off between high negative predictive values and lower positive predictive values. The regression models focused on person‐mean burnout and did not explicitly model within‐person trajectories. A precise estimation of the minimal clinically important difference for OLBI‐MS‐11 was beyond the scope of this study.

Future research could build on these findings by conducting multi‐site, cross‐cohort studies, integrating richer measures of the learning environment (e.g., workload, night duties, supervisor ratios, team culture, and sleep), embedding the OLBI‐MS‐11 in intervention trials (e.g., psychological flexibility training and mistreatment‐reduction programmes) to test sensitivity to change, and expanding anchors to include patient safety outcomes and academic performance.

## 6 Conclusions

Among Japanese medical students undertaking clinical clerkships, the OLBI‐MS short form showed good responsiveness and concurrent discrimination with coherent associations with related psychological constructs and behavioural indicators of absence. These properties were broadly consistent across academic cohorts within the same institution and across the 11‐ and 16‐item versions, suggesting that OLBI‐MS‐11 may be useful for repeated monitoring and programme evaluation in medical education, although its applicability in other educational contexts remains to be established. In practice, such monitoring may help educators and programme leaders identify sustained burnout risks during clinical clerkships and support timely educational or institutional interventions.

## Author Contributions

T.W. was responsible for conceptualisation, data curation, formal analysis, funding acquisition, and project administration. T.W. and O.T. conducted the investigation and collected data. T.A. provided overall supervision. T.W. drafted the original manuscript. O.T. and T.A. reviewed and edited it. All authors have approved the final version of the manuscript and agreed to be accountable for all aspects of this work.

## Conflicts of Interest

The authors declare no conflicts of interest.

## Supporting information


**Figure S1:** Change–change correlations between ΔOLBI‐MS‐11 and Δ external measures. Points represent Pearson's *r* with 95% confidence intervals for exhaustion and disengagement.

**Figure S2:** Interval standardised response means (SRMs) for OLBI‐MS‐11 exhaustion and disengagement. SRMs (bootstrap 95% confidence intervals; 2,000 resamples) are shown for t2–t1, t3–t2, t4–t3, and t5–t4.

**Figure S3:** Wave‐wise means with 95% confidence intervals for six indicators: OLBI‐MS‐11 Exhaustion, OLBI‐MS‐11 Disengagement and mistreatment (top row), and MBI‐EX, MBI‐CY, and absence days (bottom row). Error bars show *t*‐based 95% confidence intervals; x‐axis labels denote Waves 1–5.

**Figure S4:** Wave‐wise means all measures have 95% confidence intervals, including OLBI‐MS‐11 and OLBI‐MS‐16 subscales, MBI subscales, WAAQ, VQ, PHQ‐9, PDDS, mistreatment, and absenteeism indicators (where available).

**Figure S5:** Wave‐specific AUCs for the 16‐item OLBI‐MS. Line plot of AUC values (points with 95% DeLong confidence intervals) for OLBI‐Exhaustion → MBI‐EX > 4.0 and OLBI‐Disengagement → MBI‐CY > 2.6. Discrimination was consistently in the “good” range across waves, paralleling the 11‐item findings.

**Figure S6:** Interval SRMs for the 16‐item OLBI‐MS. Forest‐style plot of SRMs (points with bootstrap 95% confidence intervals) for OLBI‐Exhaustion and OLBI‐Disengagement by interval. Exhaustion shows small‐to‐moderate decreases (improvement), and disengagement shows small increases, mirroring the short‐form results.

**Table S1:** Full Δ–Δ correlation matrix with external measures.
**Table S2:** Level–level associations with within‐wave cumulative absence.
**Table S3:** Level/binary absence analyses (any absence in the interval = 0/1).
**Table S4:** Anchor‐based responsiveness: group summaries (Improved vs Non‐Improved).
**Table S5:** Δ‐based ROC for MBI‐defined improvement (Improved vs Non‐Improved).
**Table S6:** Wave‐wise means 95% CIs for all indicators (W1–W5).
**Table S7:** Mixed‐model tests for wave effects.
**Table S8:** Baseline predictors of person‐mean OLBI‐MS‐11 Exhaustion and Dis during clerkships (HC3‐robust OLS).
**Table S9:** Primary change–change correlations by cohort with Fisher's r‐to‐z test.
**Table S10:** AUCs by cohort with DeLong test for AUC difference.
**Table S11:** t1→t5 distribution‐based responsiveness by cohort (SRM/ES).
**Table S12:** Primary change–change correlations (16‐item OLBI‐MS).
**Table S13:** Wave‐specific and pooled AUCs for OLBI‐MS‐16 scores classifying concurrent MBI‐GS caseness.
**Table S14:** Distribution‐based responsiveness (SRM/ES) for the 16‐item OLBI‐MS.

## Data Availability

Due to ethical restrictions and participant privacy, the data are not publicly available. De‐identified data may be available from the corresponding author upon reasonable request and with appropriate approvals.
