# Supplementary figures and images for: Responsiveness of the Oldenburg Burnout Inventory for Medical Students and Predictors of Sustained Burnout during Clinical Clerkships: A Five‐Wave Longitudinal Cohort Study
Source: J Eval Clin Pract. 2026 Jun 16;32(4):e70500. doi: 10.1111/jep.70500 (PMC13271536; doi:10.1111/jep.70500)

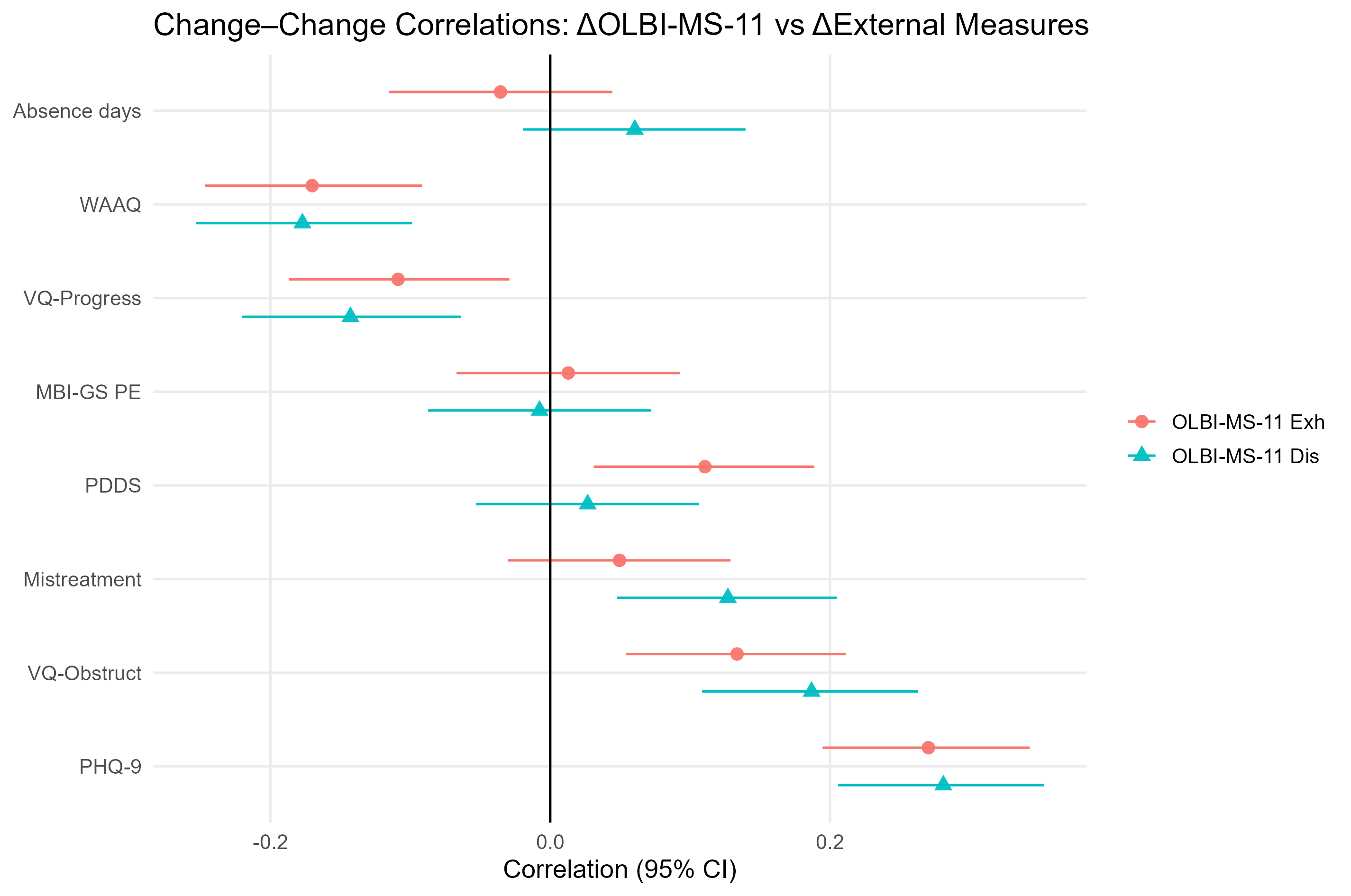

Supplement: Supplementary file 1 — Figure S1: Change–change correlations between ΔOLBI‐MS‐11 and Δ external measures. Points represent Pearson's r with 95% confidence intervals for exhaustion and disengagement. [file JEP-32-0-s005.png]

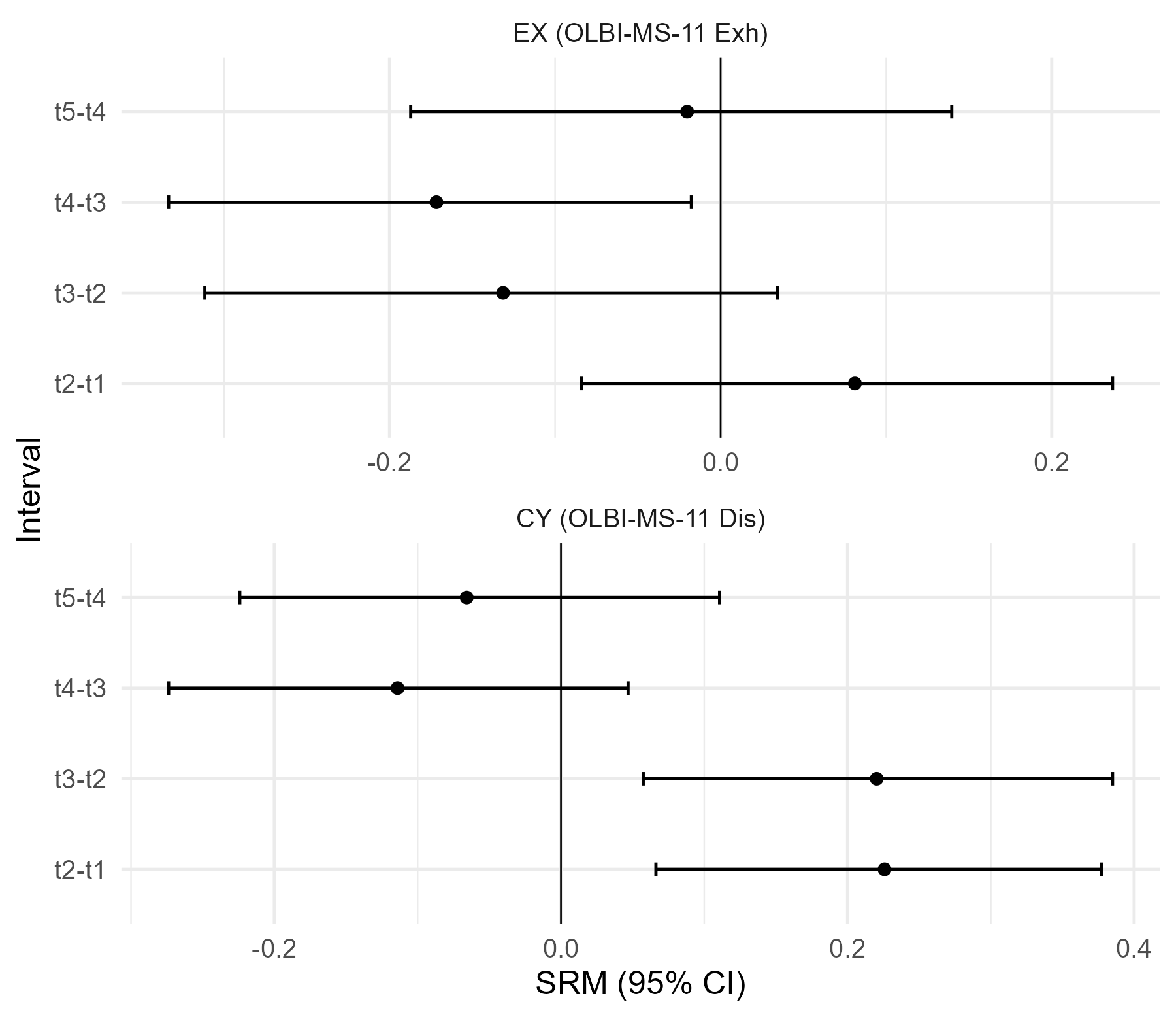

Supplement: Supplementary file 2 — Figure S2: Interval standardised response means (SRMs) for OLBI‐MS‐11 exhaustion and disengagement. SRMs (bootstrap 95% confidence intervals; 2,000 resamples) are shown for t2–t1, t3–t2, t4–t3, and t5–t4. [file JEP-32-0-s007.png]

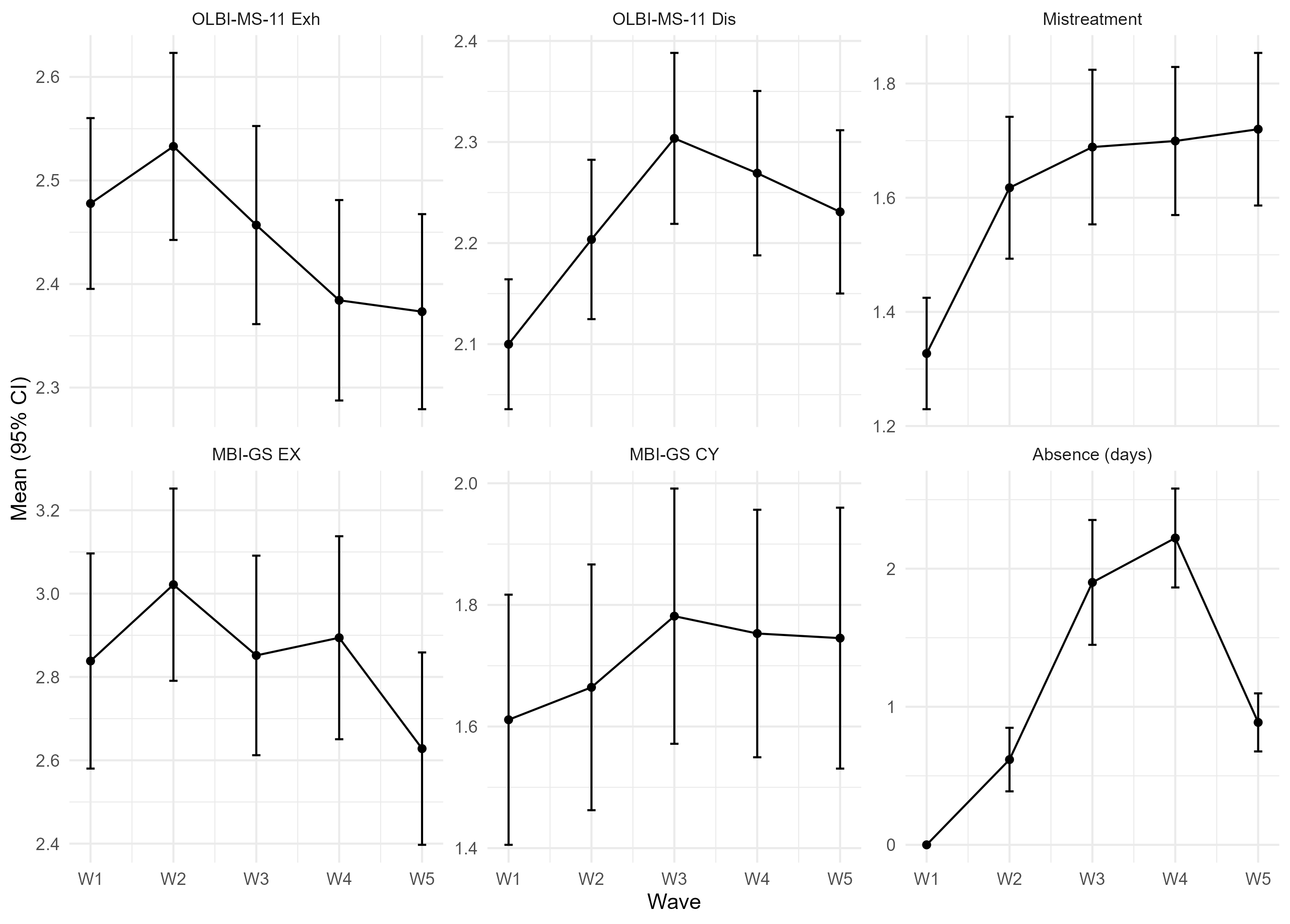

Supplement: Supplementary file 3 — Figure S3: Wave‐wise means with 95% confidence intervals for six indicators: OLBI‐MS‐11 Exhaustion, OLBI‐MS‐11 Disengagement and mistreatment (top row), and MBI‐EX, MBI‐CY, and absence days (bottom row). Error bars show t‐based 95% confidence intervals; x‐axis labels denote Waves 1–5. [file JEP-32-0-s003.png]

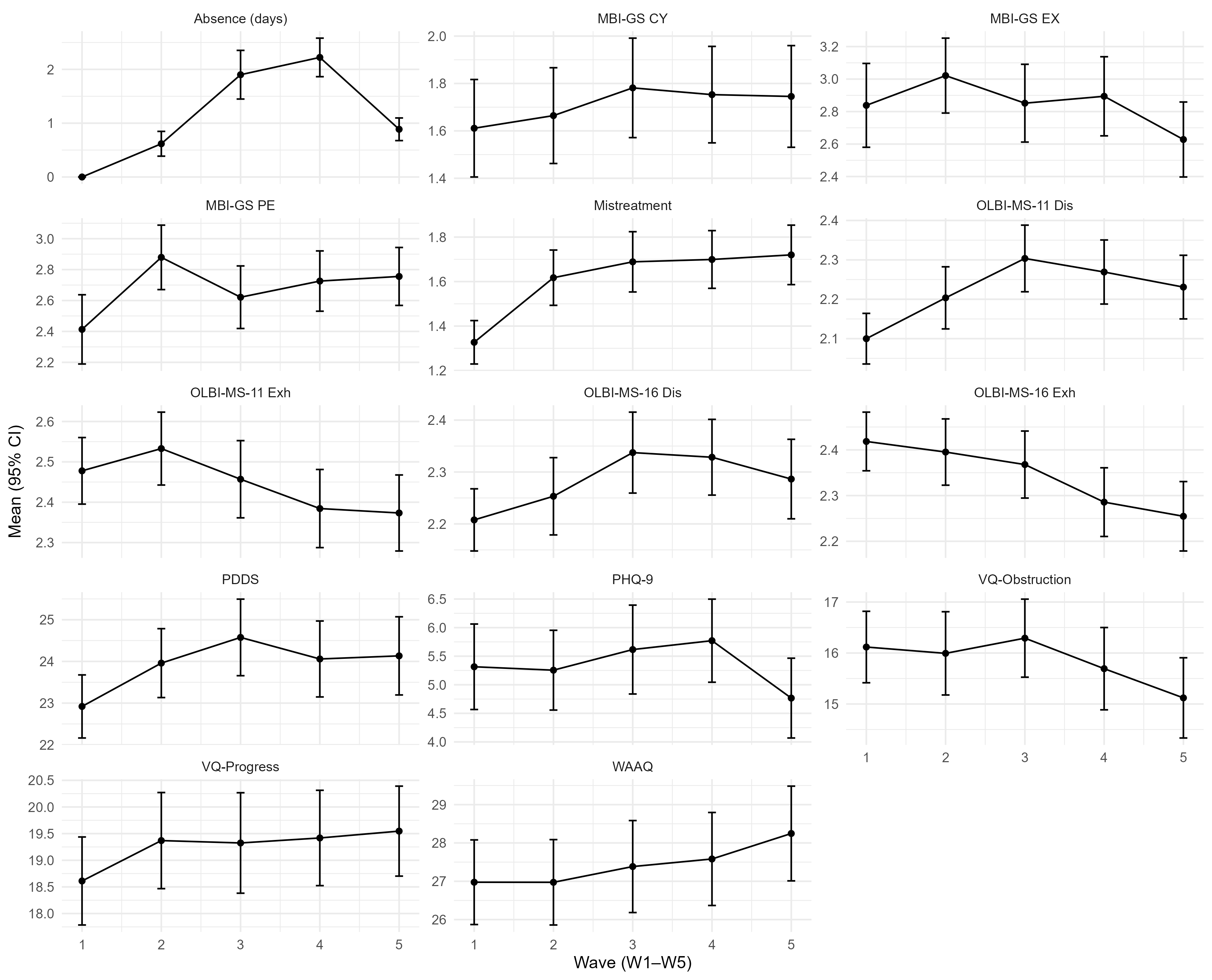

Supplement: Supplementary file 4 — Figure S4: Wave‐wise means all measures have 95% confidence intervals, including OLBI‐MS‐11 and OLBI‐MS‐16 subscales, MBI subscales, WAAQ, VQ, PHQ‐9, PDDS, mistreatment, and absenteeism indicators (where available). [file JEP-32-0-s002.png]

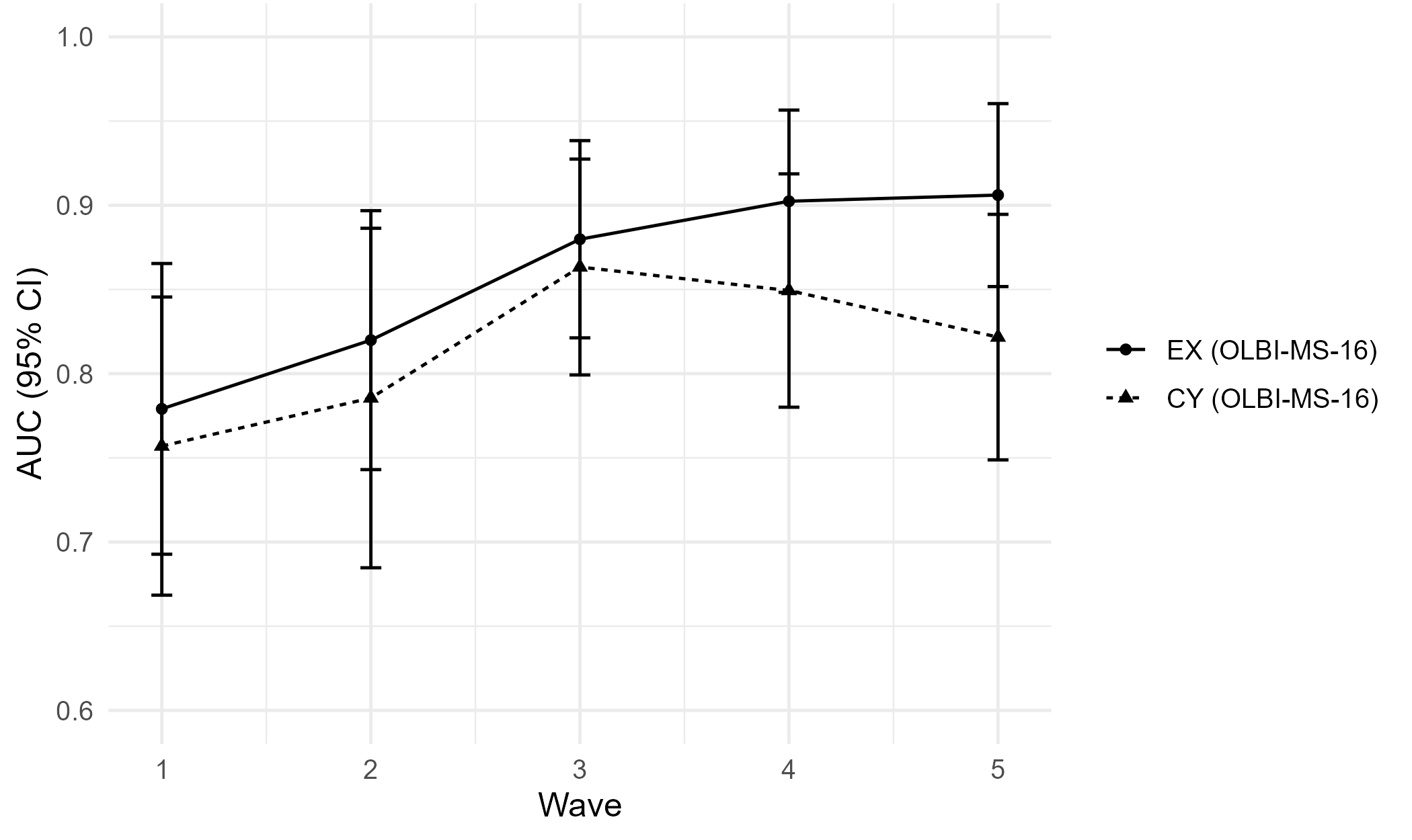

Supplement: Supplementary file 5 — Figure S5: Wave‐specific AUCs for the 16‐item OLBI‐MS. Line plot of AUC values (points with 95% DeLong confidence intervals) for OLBI‐Exhaustion → MBI‐EX > 4.0 and OLBI‐Disengagement → MBI‐CY > 2.6. Discrimination was consistently in the “good” range across waves, paralleling the 11‐item findings. [file JEP-32-0-s006.png]

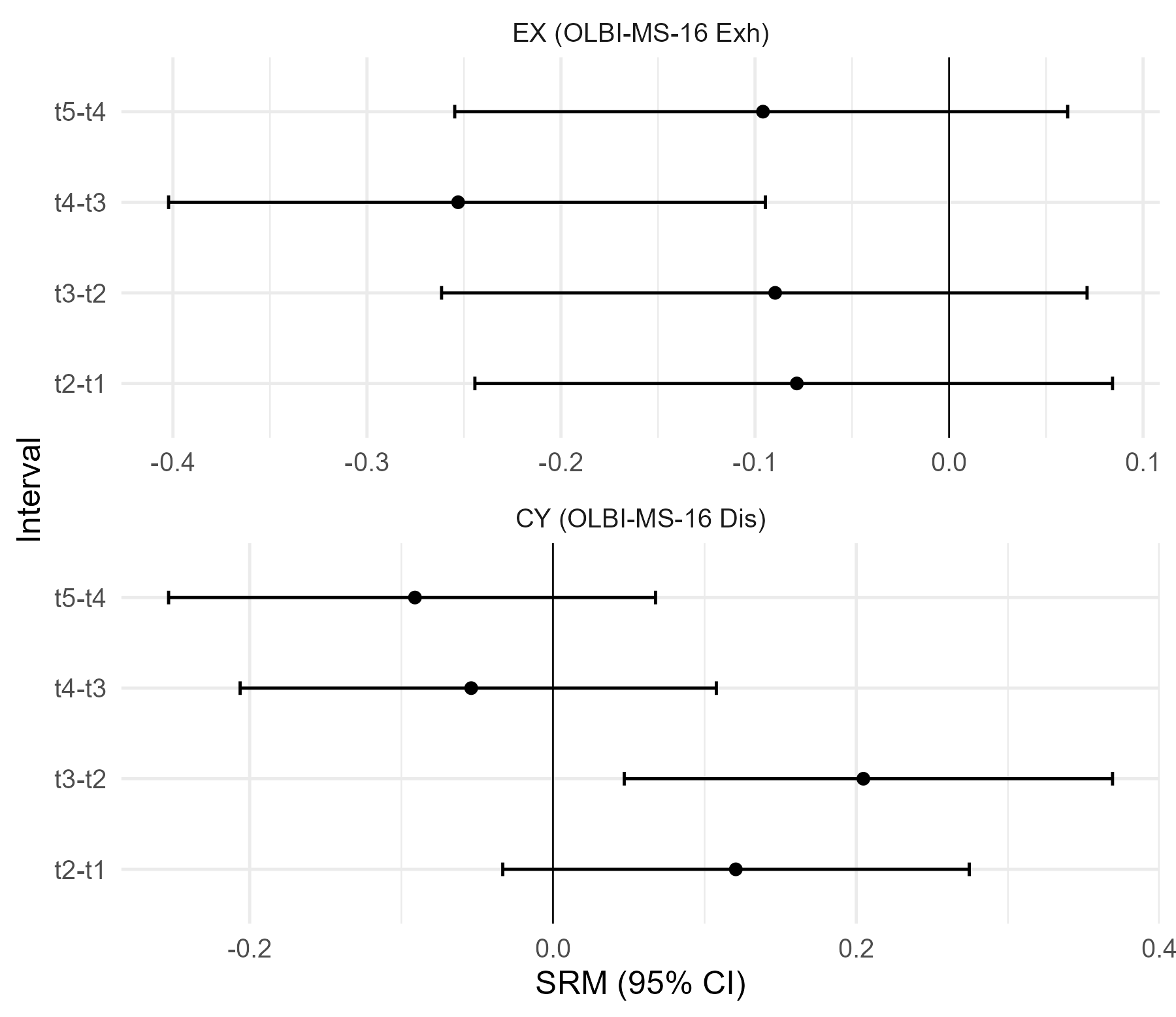

Supplement: Supplementary file 6 — Figure S6: Interval SRMs for the 16‐item OLBI‐MS. Forest‐style plot of SRMs (points with bootstrap 95% confidence intervals) for OLBI‐Exhaustion and OLBI‐Disengagement by interval. Exhaustion shows small‐to‐moderate decreases (improvement), and disengagement shows small increases, mirroring the short‐form results. [file JEP-32-0-s004.png]
