# Supplementary material for: Responsiveness of the Oldenburg Burnout Inventory for Medical Students and Predictors of Sustained Burnout during Clinical Clerkships: A Five‐Wave Longitudinal Cohort Study
Source: J Eval Clin Pract. 2026 Jun 16;32(4):e70500. doi: 10.1111/jep.70500 (PMC13271536; doi:10.1111/jep.70500)
Supplement: Supplementary file 7 — Table S1: Full Δ–Δ correlation matrix with external measures. Table S2: Level–level associations with within‐wave cumulative absence. Table S3: Level/binary absence analyses (any absence in the interval = 0/1). Table S4: Anchor‐based responsiveness: group summaries (Improved vs Non‐Improved). Table S5: Δ‐based ROC for MBI‐defined improvement (Improved vs Non‐Improved). Table S6: Wave‐wise means 95% CIs for all indicators (W1–W5). Table S7: Mixed‐model tests for wave effects. Table S8: Baseline predictors of person‐mean OLBI‐MS‐11 Exhaustion and Dis during clerkships (HC3‐robust OLS). Table S9: Primary change–change correlations by cohort with Fisher's r‐to‐z test. Table S10: AUCs by cohort with DeLong test for AUC difference. Table S11: t1→t5 distribution‐based responsiveness by cohort (SRM/ES). Table S12: Primary change–change correlations (16‐item OLBI‐MS). Table S13: Wave‐specific and pooled AUCs for OLBI‐MS‐16 scores classifying concurrent MBI‐GS caseness. Table S14: Distribution‐based responsiveness (SRM/ES) for the 16‐item OLBI‐MS. [file JEP-32-0-s001.docx]

**Supplementary Table S1. Full Δ–Δ correlation matrix with external measures**

| External measure | Subscale | n | Pearson r [95% CI] | *p*-value | Spearman ρ [95% CI] | *p*-value | Hypothesis | Direction  (Pearson) |
| --- | --- | --- | --- | --- | --- | --- | --- | --- |
| MBI-PE | Exh | 603 | 0.013 [−0.067, 0.093] | 0.751 | 0.013 [−0.067, 0.093] | 0.744 | − | No |
|  | Dis | 603 | −0.008 [−0.087, 0.072] | 0.852 | 0.009 [−0.071, 0.089] | 0.827 | − | Yes |
| WAAQ | Exh | 603 | −0.170 [−0.247, −0.092] | <0.001 | −0.142 [−0.219, −0.063] | <0.001 | − | Yes |
|  | Dis | 603 | −0.177 [−0.253, −0.099] | <0.001 | −0.147 [−0.224, −0.068] | <0.001 | − | Yes |
| VQ-P | Exh | 603 | −0.109 [−0.187, −0.029] | 0.008 | −0.110 [−0.188, −0.030] | 0.007 | − | Yes |
|  | Dis | 603 | −0.143 [−0.220, −0.064] | <0.001 | −0.110 [−0.189, −0.031] | 0.007 | − | Yes |
| VQ-O | Exh | 603 | 0.134 [0.054, 0.211] | 0.001 | 0.082 [0.002, 0.161] | 0.044 | + | Yes |
|  | Dis | 603 | 0.187 [0.109, 0.263] | <0.001 | 0.200 [0.123, 0.276] | <0.001 | + | Yes |
| PDDS | Exh | 603 | 0.111 [0.031, 0.189] | 0.007 | 0.112 [0.032, 0.190] | 0.006 | + | Yes |
|  | Dis | 603 | 0.027 [−0.053, 0.106] | 0.511 | 0.019 [−0.061, 0.098] | 0.647 | + | Yes |
| PHQ-9 | Exh | 603 | 0.270 [0.195, 0.343] | <0.001 | 0.232 [0.155, 0.306] | <0.001 | + | Yes |
|  | Dis | 603 | 0.281 [0.206, 0.353] | <0.001 | 0.261 [0.185, 0.334] | <0.001 | + | Yes |
| Mistreatment | Exh | 603 | 0.050 [−0.030, 0.129] | 0.224 | 0.026 [−0.054, 0.106] | 0.522 | + | Yes |
|  | Dis | 603 | 0.127 [0.048, 0.205] | 0.002 | 0.098 [0.018, 0.177] | 0.016 | + | Yes |
| Absence (days) | Exh | 603 | −0.036 [−0.115, 0.044] | 0.384 | −0.037 [−0.116, 0.043] | 0.364 | + | No |
|  | Dis | 603 | 0.060 [−0.019, 0.140] | 0.138 | 0.046 [−0.034, 0.126] | 0.256 | + | Yes |

Δ denotes within-person adjacent change computed as Δ = W(t) − W(t−1) for intervals W2–W1, W3–W2, W4–W3, and W5–W4; correlations pool pairwise-complete adjacent pairs (n shown per row). Primary statistic is Pearson’s r with 95% CIs via Fisher’s z; Spearman’s ρ is a sensitivity analysis. Two-sided tests; α = 0.05; no multiplicity adjustment. Hypothesis signs: negative (−) for MBI-PE, WAAQ, VQ-P; positive (+) for VQ-O, PDDS, PHQ-9, mistreatment frequency, absence. As Δabsence showed only small or non-significant associations with ΔOLBI-MS-11, detailed level-based／binary absence analyses are provided in Supplementary Tables S4–S5.

Abbreviations. OLBI-MS-11, Oldenburg Burnout Inventory—Medical Student (11-item short form); Exh, Exhaustion; Dis, Disengagement; MBI-GS, Maslach Burnout Inventory—General Survey; MBI-PE, Personal Efficacy; WAAQ, Work-related Acceptance and Action Questionnaire; VQ-P, Valuing Questionnaire—Progress; VQ-O, Valuing Questionnaire—Obstruction; PDDS, Perceived Devaluation–Discrimination Scale; PHQ-9, Patient Health Questionnaire-9; CI, confidence interval.

**Supplementary Table S2. Level–level associations with within-wave cumulative absence**

| Subscale | N | Pearson r [95% CI] | *p*-value | Spearman ρ [95% CI] | *p*-value |
| --- | --- | --- | --- | --- | --- |
| OLBI-MS-11 Exh | 765 | −0.053 [−0.123, 0.018] | 0.143 | −0.061 [−0.131, 0.010] | 0.093 |
| OLBI-MS-11 Dis | 765 | 0.058 [−0.013, 0.128] | 0.111 | 0.091 [0.020, 0.161] | 0.012 |

“Level–level” denotes within-wave (contemporaneous) associations between OLBI-MS-11 subscale levels and cumulative absence. Cumulative absence is the per-participant cumulative sum of absence days up to each wave (W1–W5), abstracted from administrative records. Correlations pool pairwise-complete wave-level observations (N shown per row). Primary statistic is Pearson’s r with 95% CIs derived via Fisher’s z; Spearman’s ρ is provided as a sensitivity analysis. Two-sided tests; α = 0.05; no multiplicity adjustment.

Abbreviations. OLBI-MS-11, Oldenburg Burnout Inventory—Medical Student (11-item short form); Exh, Exhaustion; Dis, Disengagement; CI, confidence interval.

**Supplementary Table S3. Level/binary absence analyses (any absence in the interval = 0/1).**

**(A) Correlations (level vs binary absence)**

| Subscale | N | Pearson r [95% CI] | *p*-value | Spearman ρ [95% CI] | *p*-value |
| --- | --- | --- | --- | --- | --- |
| OLBI-MS-11 Exh | 765 | −0.071 [−0.141, 0.000] | 0.051 | −0.064 [−0.134, 0.007] | 0.077 |
| OLBI-MS-11 Dis | 765 | 0.072 [0.001, 0.142] | 0.047 | 0.082 [0.011, 0.152] | 0.024 |

**(B) Group means by absence status and Welch’s t test**

| Subscale | Absence=0: N | Absence=0: Mean | Absence=1: N | Absence=1: Mean | Difference (0−1) [95% CI] | *p*-value |
| --- | --- | --- | --- | --- | --- | --- |
| OLBI-MS-11 Exh | 448 | 2.48 | 317 | 2.40 | 0.08  [−0.00, 0.17] | 0.054 |
| OLBI-MS-11 Dis | 448 | 2.19 | 317 | 2.26 | −0.07  [−0.14, −0.00] | 0.049 |

“Binary absence” indicates any absence days during the interval (coded 1) vs none (coded 0). Panel (A) reports contemporaneous correlations between OLBI-MS-11 subscale levels and the binary absence indicator at each wave; Pearson’s r is primary with 95% CIs via Fisher’s z, and Spearman’s ρ is shown as a sensitivity analysis. Panel (B) compares subscale means between absence groups using Welch’s two-sample t test; the difference is reported as mean(Absence=0) − mean(Absence=1) with 95% CIs. Analyses use pairwise-complete wave-level observations; two-sided tests, α = 0.05; no multiplicity adjustment.

Abbreviations. OLBI-MS-11, Oldenburg Burnout Inventory—Medical Student (11-item short form); Exh, Exhaustion; Dis, Disengagement; CI, confidence interval.

**Supplementary Table S4.** **Anchor-based responsiveness: group summaries (Improved vs Non-Improved)**

**(A) OLBI-MS-11 Exhaustion (MBI-EX anchor)**

| Contrast | n (Non-Improved) | Mean Δ | SD(Δ) | SRM | ES | n (Improved) | Mean Δ | SD(Δ) | SRM | ES |
| --- | --- | --- | --- | --- | --- | --- | --- | --- | --- | --- |
| t2–t1 | 135 | 0.067 | 0.553 | 0.120 | 0.123 | 14 | −0.171 | 0.429 | −0.400 | −0.358 |
| t3–t2 | 124 | −0.021 | 0.553 | −0.038 | −0.037 | 18 | −0.433 | 0.451 | −0.961 | −1.150 |
| t4–t3 | 137 | −0.058 | 0.449 | −0.130 | −0.101 | 10 | −0.360 | 0.532 | −0.677 | −0.628 |
| t5–t4 | 134 | 0.015 | 0.402 | 0.037 | 0.025 | 12 | −0.267 | 0.375 | −0.711 | −0.632 |
| Pooled | 530 | 0.001 | 0.493 | 0.002 | 0.001 | 54 | −0.315 | 0.446 | −0.706 | −0.686 |

**(B) OLBI-MS-11 Disengagement (MBI-CY anchor)**

| Contrast | n (Non-Improved) | Mean Δ | SD(Δ) | SRM | ES | n (Improved) | Mean Δ | SD(Δ) | SRM | ES |
| --- | --- | --- | --- | --- | --- | --- | --- | --- | --- | --- |
| t2–t1 | 135 | 0.067 | 0.553 | 0.120 | 0.123 | 14 | −0.171 | 0.429 | −0.400 | −0.358 |
| t3–t2 | 124 | −0.021 | 0.553 | −0.038 | −0.037 | 18 | −0.433 | 0.451 | −0.961 | −1.150 |
| t4–t3 | 137 | −0.058 | 0.449 | −0.130 | −0.101 | 10 | −0.360 | 0.532 | −0.677 | −0.628 |
| t5–t4 | 134 | 0.015 | 0.402 | 0.037 | 0.025 | 12 | −0.267 | 0.375 | −0.711 | −0.632 |
| Pooled | 530 | 0.001 | 0.493 | 0.002 | 0.001 | 54 | −0.315 | 0.446 | −0.706 | −0.686 |

Δ denotes within-person adjacent change (Δ = W(t) − W(t−1); W2–W1, W3–W2, W4–W3, W5–W4). Groups are defined by MBI anchors (EX for Exhaustion; CY for Cynicism) into Improved vs Non-Improved as specified in the Methods. SRM = mean Δ / SD(Δ). ES = mean Δ / baseline SD of the corresponding subscale within the contrast. “Pooled” aggregates all adjacent intervals. Two-sided analyses; no multiplicity adjustment.

Abbreviations. OLBI-MS-11, Oldenburg Burnout Inventory—Medical Student (11-item short form); Exh, Exhaustion; Dis, Disengagement; MBI-GS, Maslach Burnout Inventory—General Survey; EX, Exhaustion; CY, Cynicism; SRM, standardized response mean; ES, effect size.

**Supplementary** **Table S5. Δ-based ROC for MBI-defined improvement (Improved vs Non-Improved)**

| Anchor (target) | n | Improved | Non-Improved | AUC  [95% CI] | Youden-optimal Δ cut | Sensitivity | Specificity | PPV | NPV |
| --- | --- | --- | --- | --- | --- | --- | --- | --- | --- |
| EX anchor (Improved  vs Non-Improved) | 584 | 54 | 530 | 0.679  [0.603–0.755] | −0.30 | 0.54 | 0.75 | 0.18 | 0.94 |
| CY anchor (Improved  vs Non-Improved) | 584 | 55 | 529 | 0.663  [0.588–0.738] | −0.08 | 0.58 | 0.66 | 0.15 | 0.94 |

ROC analyses classify MBI-defined improvement (Improved=1 vs Non-Improved=0) using adjacent-interval ΔOLBI-MS-11 (Δ = W(t) − W(t−1)). For computational alignment with improvement (lower OLBI indicates improvement), the ROC predictor was set to −Δ; the table reports the Youden-optimal cut back-transformed into Δ units (negative values denote decreases in OLBI). AUCs are shown with DeLong 95% CIs; operating characteristics (sensitivity, specificity, PPV, NPV) are evaluated at the Youden-optimal Δ cut and are prevalence-dependent (class imbalance yields high NPV and modest PPV). Analyses pool adjacent intervals with pairwise-complete observations; two-sided tests, α = 0.05; no multiplicity adjustment.

Abbreviations. OLBI-MS-11, Oldenburg Burnout Inventory—Medical Student (11-item short form); EX, Exhaustion (MBI-GS subscale); CY, Cynicism (MBI-GS subscale); ROC, receiver operating characteristic; AUC, area under the ROC curve; CI, confidence interval; PPV, positive predictive value; NPV, negative predictive value.

**Table S6. Wave-wise means with 95% CIs for all indicators (W1–W5)**

**MBI-EX**

| Wave | n | Mean | SD | 95% CI |
| --- | --- | --- | --- | --- |
| W1 | 162 | 2.840 | 1.660 | [2.580, 3.100] |
| W2 | 149 | 3.020 | 1.420 | [2.790, 3.250] |
| W3 | 151 | 2.850 | 1.490 | [2.610, 3.090] |
| W4 | 153 | 2.890 | 1.520 | [2.650, 3.140] |
| W5 | 150 | 2.630 | 1.430 | [2.400, 2.860] |

**MBI-CY**

| Wave | n | Mean | SD | 95% CI |
| --- | --- | --- | --- | --- |
| W1 | 162 | 1.610 | 1.330 | [1.400, 1.820] |
| W2 | 149 | 1.660 | 1.250 | [1.460, 1.870] |
| W3 | 151 | 1.780 | 1.300 | [1.570, 1.990] |
| W4 | 153 | 1.750 | 1.270 | [1.550, 1.960] |
| W5 | 150 | 1.740 | 1.330 | [1.530, 1.960] |

**MBI-PE**

| Wave | n | Mean | SD | 95% CI |
| --- | --- | --- | --- | --- |
| W1 | 162 | 2.410 | 1.440 | [2.190, 2.640] |
| W2 | 149 | 2.880 | 1.290 | [2.670, 3.090] |
| W3 | 151 | 2.620 | 1.260 | [2.420, 2.820] |
| W4 | 153 | 2.730 | 1.220 | [2.530, 2.920] |
| W5 | 150 | 2.760 | 1.160 | [2.570, 2.940] |

**OLBI-MS-11 Exhaustion**

| Wave | n | Mean | SD | 95% CI |
| --- | --- | --- | --- | --- |
| W1 | 162 | 2.480 | 0.531 | [2.400, 2.560] |
| W2 | 149 | 2.530 | 0.558 | [2.440, 2.620] |
| W3 | 151 | 2.460 | 0.595 | [2.360, 2.550] |
| W4 | 153 | 2.380 | 0.606 | [2.290, 2.480] |
| W5 | 150 | 2.370 | 0.584 | [2.280, 2.470] |

**OLBI-MS-11 Disengagement**

| Wave | n | Mean | SD | 95% CI |
| --- | --- | --- | --- | --- |
| W1 | 162 | 2.100 | 0.414 | [2.040, 2.160] |
| W2 | 149 | 2.200 | 0.487 | [2.120, 2.280] |
| W3 | 151 | 2.300 | 0.526 | [2.220, 2.390] |
| W4 | 153 | 2.270 | 0.509 | [2.190, 2.350] |
| W5 | 150 | 2.230 | 0.501 | [2.150, 2.310] |

**PHQ-9**

| Wave | n | Mean | SD | SE | 95% CI |
| --- | --- | --- | --- | --- | --- |
| W1 | 162 | 5.320 | 4.820 | 0.379 | [4.570, 6.060] |
| W2 | 149 | 5.260 | 4.310 | 0.353 | [4.560, 5.950] |
| W3 | 151 | 5.620 | 4.840 | 0.394 | [4.840, 6.390] |
| W4 | 153 | 5.770 | 4.550 | 0.368 | [5.040, 6.500] |
| W5 | 150 | 4.770 | 4.330 | 0.353 | [4.070, 5.460] |

**VQ-Progress (VQ-P)**

| Wave | n | Mean | SD | SE | 95% CI |
| --- | --- | --- | --- | --- | --- |
| W1 | 162 | 18.600 | 5.320 | 0.418 | [17.800, 19.400] |
| W2 | 149 | 19.400 | 5.570 | 0.456 | [18.500, 20.300] |
| W3 | 151 | 19.300 | 5.860 | 0.477 | [18.400, 20.300] |
| W4 | 153 | 19.400 | 5.600 | 0.453 | [18.500, 20.300] |
| W5 | 150 | 19.500 | 5.230 | 0.427 | [18.700, 20.400] |

**VQ-Obstruction (VQ-O)**

| Wave | n | Mean | SD | SE | 95% CI |
| --- | --- | --- | --- | --- | --- |
| W1 | 162 | 16.100 | 4.520 | 0.355 | [15.400, 16.800] |
| W2 | 149 | 16.000 | 5.040 | 0.413 | [15.200, 16.800] |
| W3 | 151 | 16.300 | 4.760 | 0.387 | [15.500, 17.100] |
| W4 | 153 | 15.700 | 5.050 | 0.408 | [14.900, 16.500] |
| W5 | 150 | 15.100 | 4.880 | 0.398 | [14.300, 15.900] |

**WAAQ**

| Wave | n | Mean | SD | SE | 95% CI |
| --- | --- | --- | --- | --- | --- |
| W1 | 162 | 27.000 | 7.120 | 0.559 | [25.900, 28.100] |
| W2 | 149 | 27.000 | 6.880 | 0.564 | [25.900, 28.100] |
| W3 | 151 | 27.400 | 7.460 | 0.607 | [26.200, 28.600] |
| W4 | 153 | 27.600 | 7.600 | 0.614 | [26.400, 28.800] |
| W5 | 150 | 28.200 | 7.660 | 0.625 | [27.000, 29.500] |

**PDDS**

| Wave | n | Mean | SD | SE | 95% CI |
| --- | --- | --- | --- | --- | --- |
| W1 | 162 | 22.900 | 4.880 | 0.383 | [22.200, 23.700] |
| W2 | 149 | 24.000 | 5.110 | 0.418 | [23.100, 24.800] |
| W3 | 151 | 24.600 | 5.710 | 0.465 | [23.700, 25.500] |
| W4 | 153 | 24.100 | 5.700 | 0.461 | [23.100, 25.000] |
| W5 | 150 | 24.100 | 5.810 | 0.474 | [23.200, 25.100] |

**Mistreatment**

| Wave | n | Mean | SD | SE | 95% CI |
| --- | --- | --- | --- | --- | --- |
| W1 | 162 | 1.330 | 0.629 | 0.049 | [1.230, 1.420] |
| W2 | 149 | 1.620 | 0.768 | 0.063 | [1.490, 1.740] |
| W3 | 151 | 1.690 | 0.842 | 0.069 | [1.550, 1.820] |
| W4 | 153 | 1.700 | 0.812 | 0.066 | [1.570, 1.830] |
| W5 | 150 | 1.720 | 0.828 | 0.068 | [1.590, 1.850] |

**Absence (days)**

| Wave | n | Mean | SD | SE | 95% CI |
| --- | --- | --- | --- | --- | --- |
| W1 | 162 | 0.000 | 0.000 | 0.000 | [0.000, 0.000] |
| W2 | 149 | 0.617 | 1.420 | 0.116 | [0.387, 0.848] |
| W3 | 151 | 1.900 | 2.810 | 0.229 | [1.450, 2.350] |
| W4 | 153 | 2.220 | 2.240 | 0.181 | [1.860, 2.580] |
| W5 | 150 | 0.887 | 1.300 | 0.106 | [0.676, 1.100] |

Entries are n, mean, SD, and 95% CI by wave. Estimates are raw (non–model-based); confidence intervals use a t distribution with df = n−1.
Abbreviations: OLBI-MS-11, Oldenburg Burnout Inventory—Medical Student (11-item short form); Exh, Exhaustion; Dis, Disengagement; MBI-GS, Maslach Burnout Inventory—General Survey; EX, Exhaustion; CY, Cynicism; PE, Professional Efficacy; PHQ-9, Patient Health Questionnaire-9; WAAQ, Work-related Acceptance and Action Questionnaire; VQ-P, Valuing Questionnaire—Progress; VQ-O, Valuing Questionnaire—Obstruction; PDDS, Perceived Devaluation–Discrimination Scale; CI, confidence interval; SD, Standard Deviation.

**Table S7. Mixed-model tests for wave effects**

| Indicator | NumDF | DenDF | F | *p*-value | *p-value* (FDR) | N (obs) | N (IDs) |
| --- | --- | --- | --- | --- | --- | --- | --- |
| OLBI-MS-11 Exhaustion | 4 | 606.0 | 4.60 | 0.0011 | 0.0020 | 765 | 162 |
| OLBI-MS-11 Disengagement | 4 | 609.0 | 9.87 | <0.0001 | <0.0001 | 765 | 162 |
| OLBI-MS-16 Exhaustion | 4 | 607.0 | 9.83 | <0.0001 | <0.0001 | 765 | 162 |
| OLBI-MS-16 Disengagement | 4 | 608.0 | 5.80 | 0.0001 | 0.0003 | 765 | 162 |
| MBI-EX | 4 | 605.0 | 3.59 | 0.0066 | 0.0103 | 765 | 162 |
| MBI-CY | 4 | 608.0 | 1.00 | 0.4040 | 0.4040 | 765 | 162 |
| MBI-PE | 4 | 606.0 | 5.06 | 0.0005 | 0.0010 | 765 | 162 |
| WAAQ | 4 | 607.0 | 1.75 | 0.1370 | 0.1600 | 765 | 162 |
| VQ-Progress | 4 | 609.0 | 1.35 | 0.2480 | 0.2680 | 765 | 162 |
| VQ-Obstruction | 4 | 608.0 | 2.65 | 0.0323 | 0.0452 | 765 | 162 |
| PHQ-9 | 4 | 606.0 | 2.48 | 0.0426 | 0.0542 | 765 | 162 |
| PDDS | 4 | 607.0 | 5.88 | 0.0001 | 0.0003 | 765 | 162 |
| Mistreatment | 4 | 608.0 | 14.10 | <0.0001 | <0.0001 | 765 | 162 |
| Absence (days) | 4 | 615.0 | 45.00 | <0.0001 | <0.0001 | 765 | 162 |

Tests are for the fixed effect of wave (factor-coded) in linear mixed-effects models: *outcome* ~ factor(wave) + cohort + (1|id). Type III tests with Satterthwaite degrees of freedom (lmerTest). NumDF/DenDF = numerator/denominator degrees of freedom. p-values are unadjusted; “p-value (FDR)” are Benjamini–Hochberg false-discovery-rate–adjusted across indicators. *N (obs)* = number of wave-level observations; *N (IDs)* = unique participants. (Two-sided.)
Abbreviations: OLBI-MS-11/-16, Oldenburg Burnout Inventory—Medical Student (11-/16-item); MBI-EX/CY/PE, Maslach Burnout Inventory—General Survey (Exhaustion/Cynicism/Personal Efficacy subscales); WAAQ, Work-related Acceptance and Action Questionnaire; VQ-Progress/VQ-Obstruction (VQ-P/VQ-O); PHQ-9, Patient Health Questionnaire-9; PDDS, Perceived Devaluation–Discrimination Scale; FDR, false discovery rate.

**Table S8.** **Baseline predictors of person-mean OLBI-MS-11 Exhaustion and Dis during clerkships (HC3-robust OLS)**

(A) Outcome: OLBI-MS-11 Exhaustion (Exh)

| Predictor | β [95% CI] | *p*-value |
| --- | --- | --- |
| Intercept | 2.497 [2.376, 2.618] | <0.001 |
| Age (z) | -0.041 [-0.120, 0.037] | 0.302 |
| Gender: men | -0.090 [-0.239, 0.059] | 0.236 |
| WAAQ (z) | -0.141 [-0.216, -0.066] | <0.001 |
| WAAQ (z) | 0.027 [-0.042, 0.095] | 0.443 |
| Mistreatment (z) | 0.109 [0.038, 0.181] | 0.003 |

(B) Outcome: OLBI-MS-11 Disengagement (Dis)

| Predictor | β [95% CI] | *p*-value |
| --- | --- | --- |
| Intercept | 2.240 [2.135, 2.344] | <0.001 |
| Age (z) | -0.023 [-0.105, 0.058] | 0.570 |
| Gender (men) | -0.029 [-0.158, 0.100] | 0.657 |
| WAAQ (z) | -0.106 [-0.166, -0.046] | <0.001 |
| WAAQ (z) | 0.054 [-0.012, 0.121] | 0.108 |
| Mistreatment (z) | 0.046 [-0.005, 0.097] | 0.077 |

Ordinary least squares coefficients with HC3 robust standard errors are shown as β with 95% CIs; two-sided tests, α = 0.05. Continuous predictors were standardized (z-scores). Gender coefficient compares men vs women (reference). Outcomes are person-level means across Waves 1–5. Complete-case analysis; no imputation.
Abbreviations: OLBI-MS-11, Oldenburg Burnout Inventory—Medical Student (11-item short form); Exh, Exhaustion; Dis, Disengagement; WAAQ, Work-related Acceptance and Action Questionnaire; PDDS, Perceived Devaluation–Discrimination Scale; OLS, ordinary least squares; HC3, heteroskedasticity-consistent (type 3); CI, Confidence Interval.

**Table S9. Primary change–change correlations by cohort with Fisher’s r-to-z test**

| Pair | Cohort | n | Pearson r  [95% CI] | *p*-value |
| --- | --- | --- | --- | --- |
| ΔOLBI-MS-11 Exh  ↔ ΔMBI-EX | 2023 | 322 | 0.308  [0.206, 0.404] | 0.236 |
| ΔOLBI-MS-11 Exh  ↔ ΔMBI-EX | 2024 | 281 | 0.393  [0.290, 0.488] | 0.236 |
| ΔOLBI-MS-11 Dis  ↔ ΔMBI-CY | 2023 | 322 | 0.366  [0.267, 0.457] | 0.589 |
| ΔOLBI-MS-11 Dis ↔ ΔMBI-CY | 2024 | 281 | 0.327  [0.218, 0.427] | 0.589 |

Δ denotes within-person adjacent change computed as W(t) − W(t−1), pooled across adjacent intervals (W2–W1 through W5–W4) using pairwise-complete observations. Estimates are Pearson’s r with 95% CI from Fisher’s z transformation. The reported p value tests the difference between cohort-specific correlations via Fisher’s r-to-z (two-sided, α = 0.05; no multiplicity adjustment). Abbreviations: OLBI-MS-11, Oldenburg Burnout Inventory—Medical Student (11-item short form); Exh, Exhaustion; Dis, Disengagement; MBI-GS, Maslach Burnout Inventory—General Survey; EX, Exhaustion; CY, Cynicism; CI, confidence interval.

**Table S10. AUCs by cohort with DeLong test for AUC difference**

| Target | Cohort | N | Positives | Negatives | AUC  [95% CI] | DeLong *p*-value |
| --- | --- | --- | --- | --- | --- | --- |
| OLBI-MS-11 Exh  → MBI-EX caseness | 2023 | 410 | 97 | 313 | 0.864  [0.823, 0.904] | 0.250 |
| OLBI-MS-11 Exh  → MBI-EX caseness | 2024 | 355 | 81 | 274 | 0.826  [0.776, 0.875] | 0.250 |
| OLBI-MS-11 Dis  → MBI-CY caseness | 2023 | 410 | 90 | 320 | 0.812  [0.765, 0.859] | 0.605 |
| OLBI-MS-11 Dis  → MBI-CY caseness | 2024 | 355 | 79 | 276 | 0.830  [0.779, 0.882] | 0.605 |

Caseness anchors followed prior literature: MBI-EX > 4.0 and MBI-CY > 2.6 (see Methods for citations). Cohort-specific AUCs are reported with 95% CIs by DeLong’s method. The DeLong *p* value tests the between-cohort AUC difference for each target (two-sided, α = 0.05; no multiplicity adjustment). N, Positives, and Negatives denote the number of available observations and caseness counts within each cohort.

Abbreviations: OLBI-MS-11, Oldenburg Burnout Inventory—Medical Student (11-item short form); Exh, Exhaustion; Dis, Disengagement; MBI-GS, Maslach Burnout Inventory—General Survey; EX, Exhaustion; CY, Cynicism; CI, confidence interval; AUC, area under the curve.

**Table S11. t1→t5 distribution-based responsiveness by cohort (SRM/ES)**

| Cohort | Scale | N | Mean (t1) | SD (t1) | Mean (t5) | SD (t5) | Mean Δ (t5–t1) | SD(Δ) | SRM  [95% CI] | ES  [95% CI] |
| --- | --- | --- | --- | --- | --- | --- | --- | --- | --- | --- |
| 2023 | OLBI-MS-11  Exh | 80 | 2.430 | 0.534 | 2.380 | 0.601 | −0.052 | 0.576 | −0.091  [−0.307, 0.124] | −0.098  [−0.337, 0.130] |
| 2023 | OLBI-MS-11  Dis | 80 | 2.050 | 0.398 | 2.250 | 0.528 | 0.200 | 0.505 | 0.396  [ 0.154, 0.660] | 0.503  [ 0.209, 0.814] |
| 2024 | OLBI-MS-11  Exh | 70 | 2.530 | 0.520 | 2.370 | 0.567 | −0.160 | 0.593 | −0.270  [−0.569, −0.045] | −0.308  [−0.593, −0.051] |
| 2024 | OLBI-MS-11  Dis | 70 | 2.140 | 0.448 | 2.210 | 0.470 | 0.073 | 0.457 | 0.160  [−0.077, 0.380] | 0.163  [−0.071, 0.409] |

Contrast is t5−t1 within persons with data at both waves (complete-case). SRM = mean Δ / SD(Δ); ES = mean Δ / SD at t1. 95% CIs for SRM and ES were obtained via 2,000-iteration bootstrap.

Abbreviations: OLBI-MS-11, Oldenburg Burnout Inventory—Medical Student (11-item short form); Exh, Exhaustion; Dis, Disengagement; SRM, standardized response mean; ES, effect size; CI, confidence interval.

**Table S12. Primary change–change correlations (16-item OLBI-MS)**

| Pair* | n | Pearson r**  [95% CI] | Spearman ρ***  [95% CI] | p-value |
| --- | --- | --- | --- | --- |
| ΔOLBI-MS-16 Exh  ↔ ΔMBI-EX | 603 | 0.395  [0.325, 0.460] | 0.396  [0.327, 0.462] | <0.001 |
| ΔOLBI-MS-16 Dis  ↔ ΔMBI-CY | 603 | 0.350  [0.277, 0.418] | 0.342  [0.269, 0.410] | <0.001 |

Δ denotes within-person adjacent change computed as Δ = W(t) − W(t−1) for intervals W2–W1, W3–W2, W4–W3, and W5–W4. Pearson’s r is the primary estimate; 95% CIs are obtained via Fisher’s z transformation. Spearman’s ρ is provided as a sensitivity analysis. Two-sided tests with α = 0.05; no multiplicity adjustment. *n* reflects pairwise-complete observations pooled across all adjacent intervals. Domain-concordant anchors: ΔOLBI-MS-16 Exh with ΔMBI-EX; ΔOLBI-MS-16 Dis with ΔMBI-CY.
**Abbreviations:** OLBI-MS-16, Oldenburg Burnout Inventory—Medical Student (16-item); Exh, Exhaustion; Dis, Disengagement; MBI-GS, Maslach Burnout Inventory—General Survey; EX, Exhaustion; CY, Cynicism; CI, confidence interval.

**Table S13. Wave-specific and pooled AUCs for OLBI-MS-16 scores classifying concurrent MBI-GS caseness**

**(A) Exh → MBI-EX**

| Wave | N | Positives | Negatives | AUC [95% CI] |
| --- | --- | --- | --- | --- |
| 1 | 162 | 41 | 121 | 0.779 [0.693, 0.865] |
| 2 | 149 | 41 | 108 | 0.820 [0.743, 0.897] |
| 3 | 151 | 36 | 115 | 0.880 [0.821, 0.938] |
| 4 | 153 | 33 | 120 | 0.902 [0.848, 0.957] |
| 5 | 150 | 27 | 123 | 0.906 [0.852, 0.960] |
| Pooled | 765 | 178 | 587 | 0.855 [0.825, 0.886] |

**(B) Dis → MBI-CY**

| Wave | N | Positives | Negatives | AUC [95% CI] |
| --- | --- | --- | --- | --- |
| 1 | 162 | 32 | 130 | 0.757 [0.668, 0.846] |
| 2 | 149 | 27 | 122 | 0.786 [0.685, 0.886] |
| 3 | 151 | 39 | 112 | 0.863 [0.799, 0.927] |
| 4 | 153 | 34 | 119 | 0.849 [0.780, 0.919] |
| 5 | 150 | 37 | 113 | 0.822 [0.749, 0.895] |
| Pooled | 765 | 169 | 596 | 0.815 [0.780, 0.850] |

(A) Exh → MBI-EX, (B) Dis → MBI-CY. Caseness thresholds were pre-specified from prior literature and are detailed in *Statistical analysis*: MBI-EX > 4.0 and MBI-CY > 2.6. For each wave and for the pooled data, contemporaneous ROC curves were estimated for OLBI-MS-16 Exh against MBI-EX and for OLBI-MS-16 Dis against MBI-CY. AUCs are reported with 95% CIs by DeLong; where provided, Youden-optimal OLBI-MS-16 cut points appear with sensitivity and specificity (descriptive only). “Pooled” aggregates all wave-level observations. N denotes observations with non-missing OLBI-MS-16 and MBI-GS at that wave; Positives/Negatives are counts above/below the panel-specific MBI threshold. We interpreted AUC ≥ 0.70 as acceptable discrimination. Pooled Youden-optimal thresholds (descriptive): Exh 2.56; Dis 2.44.
Abbreviations: OLBI-MS-16, Oldenburg Burnout Inventory—Medical Student (16-item form); Exh, Exhaustion; Dis, Disengagement; MBI-GS, Maslach Burnout Inventory—General Survey; EX, Exhaustion; CY, Cynicism; CI, Confidence Interval; AUC, Area Under the Curve.

**Table S14. Distribution-based responsiveness (SRM/ES) for the 16-item OLBI-MS**

| Type | Contrast | Scale | n | SRM  [95% CI] | ES  [95% CI] |
| --- | --- | --- | --- | --- | --- |
| Adjacent | t2–t1 | OLBI-MS-16  Exh | 149 | −0.079  [−0.244, 0.084] | −0.077  [−0.236, 0.082] |
| Adjacent | t3–t2 | OLBI-MS-16  Exh | 142 | −0.090  [−0.262, 0.071] | −0.079  [−0.234, 0.065] |
| Adjacent | t4–t3 | OLBI-MS-16  Exh | 147 | −0.253  [−0.402, −0.095] | −0.187  [−0.313, −0.068] |
| Adjacent | t5–t4 | OLBI-MS-16  Exh | 146 | −0.096  [−0.255, 0.061] | −0.057  [−0.154, 0.035] |
| Adjacent | t2–t1 | OLBI-MS-16  Dis | 149 | 0.121  [−0.033, 0.274] | 0.133  [−0.036, 0.313] |
| Adjacent | t3–t2 | OLBI-MS-16  Dis | 142 | 0.205  [0.047, 0.369] | 0.170  [0.038, 0.307] |
| Adjacent | t4–t3 | OLBI-MS-16  Dis | 147 | −0.054  [−0.206, 0.108] | −0.038  [−0.151, 0.074] |
| Adjacent | t5–t4 | OLBI-MS-16  Dis | 146 | −0.091  [−0.253, 0.068] | −0.059  [−0.167, 0.041] |
| Baseline-anchored | t2–t1 | OLBI-MS-16  Exh | 149 | −0.079  [−0.242, 0.083] | −0.077  [−0.237, 0.082] |
| Baseline-anchored | t3–t1 | OLBI-MS-16  Exh | 151 | −0.120  [−0.296, 0.027] | −0.133  [−0.313, 0.031] |
| Baseline-anchored | t4–t1 | OLBI-MS-16  Exh | 153 | −0.337  [−0.514, −0.185] | −0.350  [−0.526, −0.191] |
| Baseline-anchored | t5–t1 | OLBI-MS-16  Exh | 150 | −0.353  [−0.518, −0.193] | −0.389  [−0.576, −0.216] |
| Baseline-anchored | t2–t1 | OLBI-MS-16  Dis | 149 | 0.121  [−0.043, 0.285] | 0.133  [−0.048, 0.317] |
| Baseline-anchored | t3–t1 | OLBI-MS-16  Dis | 151 | 0.294  [0.143, 0.446] | 0.356  [0.168, 0.563] |
| Baseline-anchored | t4–t1 | OLBI-MS-16  Dis | 153 | 0.282  [0.126, 0.439] | 0.316  [0.138, 0.505] |
| Baseline-anchored | t5–t1 | OLBI-MS-16  Dis | 150 | 0.206  [0.048, 0.365] | 0.232  [0.055, 0.419] |

“Adjacent” contrasts compare consecutive waves (t2–t1, t3–t2, t4–t3, t5–t4). “Baseline-anchored” contrasts compare each later wave with baseline (t2–t1, t3–t1, t4–t1, t5–t1). Change is defined as score at *t₂* minus score at *t₁*. SRM = mean change / SD of change; ES = mean change / SD at baseline for the corresponding contrast. 95% confidence intervals are bootstrap intervals based on 2,000 resamples. *n* is the number of participants with non-missing scores at both time points for each contrast; no imputation was performed.
Abbreviations: OLBI-MS-16, Oldenburg Burnout Inventory—Medical Student (16-item form); Exh, Exhaustion; Dis, Disengagement; SRM, standardized response mean; ES, effect size; CI, confidence interval.
